# A Game-Based Tool for Reducing Jargon Use by Medical Trainees

**DOI:** 10.15766/mep_2374-8265.11411

**Published:** 2024-06-07

**Authors:** Emily Burney, Megha Arora, Mizan Gaillard, Maya Herzig, Leo Lester, Su Park, Cliff Coleman

**Affiliations:** 1 Fourth-Year Medical Student, Oregon Health & Science University School of Medicine; 2 Third-Year Medical Student, Oregon Health & Science University School of Medicine; 3 Second-Year Medical Student, Oregon Health & Science University School of Medicine; 4 First-Year Resident, Department of Otolaryngology, University of New Mexico School of Medicine; 5 First-Year Resident, Department of Family Medicine, Natividad Medical Center; 6 Associate Professor and Clinical Thread Director for Professionalism, Ethics, and Communication, Office of the Dean, Oregon Health & Science University School of Medicine; Doris and Mark Storms Chair in Compassionate Communication, Center for Ethics in Healthcare

**Keywords:** Jargon, Patient Education, Communication Skills, Games, Health Literacy, Patient Care, Physician-Patient Relationship

## Abstract

**Introduction:**

Physicians can be unaware that many US adults have intermediate or lower health literacy. Avoiding medical jargon in patient communication can improve poor outcomes associated with lower health literacy, but physicians may struggle to do so as health literacy education is neither standardized nor universal at US allopathic medical schools. As with other skills-based proficiencies in medical education, repeat exposure and active learning help build competency. Medical students developed the Patient Communication Challenge (PCC), an adaptation of the Hasbro game Taboo, to facilitate practice of patient-centered communication skills among medical trainees.

**Methods:**

Hour-long workshops were held for groups of preclinical medical students. Students watched a communication exemplar video, played the PCC game, and completed a postworkshop survey. To play, two teams competed to earn points by identifying medical concepts as explained by a teammate who described the term without using medical jargon.

**Results:**

Evaluations indicated that the game was enjoyable and reinforced didactic concepts through active learning, with self-reported participant satisfaction and competency gain. Overall, 59% of participants (53 of 90) completed postworkshop surveys; 91% (48 of 53) agreed they felt more proficient in avoiding jargon, 94% (50 of 53) would recommend the workshop to a classmate, and 100% (53 of 53) would play again.

**Discussion:**

The PCC can help early medical trainees develop health communication skills through gamification with utilization of adult learning principles and adequate frequency for skill retention. Future applications include longitudinal assessment and expanding to later stages of medical training and other health professions.

## Educational Objectives

By the end of this activity, learners will be able to:

1. Apply patient-centered communication and best practices for health literacy and clear communication.
2. Practice using plain language in place of medical terminology to explain concepts to patients in a gameplay-based educational activity.
3. Replace medical jargon with plain language to explain common diagnoses, tests, and procedures in patient care settings.
4. Identify and reflect on opportunities and barriers to utilizing patient-centered plain-language communication to explain common diagnoses, tests, and procedures beyond the gameplay-based educational framework.

## Introduction

Health literacy is defined as the degree to which individuals have the capacity to obtain, process, and understand basic health information and services needed to make appropriate health decisions.^1^ However, most adults in the United States have intermediate or lower health literacy, which is associated with poorer health outcomes.^2,3^ Previous efforts to improve these outcomes have generated recommendations surrounding a universal precautions approach to be employed by health care professionals caring for patients.^4,5^ This approach includes the avoidance of medical jargon, which is medical terminology unfamiliar to those without medical backgrounds, among other patient-centered communication techniques.^6,7^ Medical jargon avoidance requires translation of medical concepts into patient-centered plain language by the health care provider throughout all provider-patient communication.^6,7^ Other work in this field has shown associations between improved health outcomes and provider-centered interventions informed by health literacy.^4^

However, health professionals often lack the knowledge or skills to address low health literacy through these interventions, as health communication education in US medical training is neither standardized nor universal.^2^ Despite national guidelines, educational methods vary (including role-play, skits, videos, and games), may not allow active learning or repeated exposure, or else must be purchased.^2,8,9^ This discrepancy extends to residency programs. Less than half of surveyed family medicine residencies reported health literacy teaching in the curriculum, and residents struggled to use clear communication skills when they learned these techniques.^10,11^ Medical students and residents need practice in addressing their patients’ low health literacy by using plain language and avoiding medical jargon. Without explicit health literacy training that alerts them to the use of jargon and helps them substitute more familiar words and phrases, physicians may be hampered in communicating clearly with their patients, especially as a number of national initiatives have highlighted the need for improving medical communication.^6,12–16^

At our institution, the health literacy and clear communication curriculum teaches students to avoid unnecessary jargon, among other best practices, and has been described elsewhere.^17^ Medical students formed a student group at our institution in 2019 to supplement this curriculum with additional opportunities to practice using plain, nonjargon language. Repeated exposure and active learning are required to build competency in health literacy and communication.^2,10,11,18,19^

Previous resources on health literacy and communication skills published in *MedEdPORTAL* include workshops held once or twice and focusing primarily on clerkship medical students and residents.^20–22^ This approach does not allow for repeated exposure and bypasses 2 years of opportunity for communication skill-building before students begin frequent patient interaction during their clinical experiences.

We created the Patient Communication Challenge (PCC) as an educational adaptation of the game Taboo that would allow for fun, repeated practice of plain-language communication.^23^ Gamification is a growing strategy in medical education to increase engagement, enjoyment, and retention.^24^ The PCC is the only known jargon-avoidance educational game using American English terminology.

A commercially available jargon-avoidance game developed for use in the United Kingdom includes jargon terms related to four medical subjects.^9^ Our game improves on this concept by focusing on jargon related to recent coursework. Held after each didactic systems-based course (block) during a week of protected time free of other educational responsibilities, the PCC workshop uses concepts learned during the block with terms drawn from exam preparation resources, thereby spiraling concepts.^9,25,26^ For example, after students complete coursework in immunology, the PCC includes terms like *vaccination* and *blood type.* Our goal was to implement the PCC, an innovative game to help medical students practice avoiding medical jargon and using plain language, to improve patient communication skills and to assess whether students found it to be an enjoyable and accessible medical education tool for practicing patient-centered communication.

## Methods

Via workshops, we offered the PCC to preclinical medical students after they had completed each didactic block. Students self-selected the PCC workshop via a first-come, first-served model as one of their three mandatory postexam events. During the academic year, 7 weeks of protected postexam time were held for both first- and second-year medical students at our institution. PCC workshops were an hour long, although facilitators required from 10 minutes to 1 hour of preparation time prior to the workshop (Appendix A). Workshops were limited to between five and 30 participants to enable optimal engagement.

One week prior to the workshop, participants watched an institution-specific video created by the authors on communication and health literacy (Appendix B).^27^ This video served as a review of topics taught in our institution's medical school curriculum, which included teaching on health literacy and clear communication best practices.^17^ During the first 10 minutes of the workshop, the facilitator reviewed health literacy concepts and communication techniques covered by the video and institutional teaching; reinforced that the goal of the PCC was practice over points, meaning prioritizing practicing communicating in ways future patients would understand over winning the game; and provided gameplay instructions via a PowerPoint presentation (Appendix C).

A medical jargon game, the PCC was inspired by the rules of the Hasbro game Taboo.^23^ In Taboo, players are divided into two teams (Teams A and B). Each team chooses one member to be the first clue-giver. Team A's clue-giver draws a concept card and describes the concept to their teammates without using the associated forbidden (taboo) words listed on the card. Members of Team A shout out their guesses, and the team wins a point if its members correctly identify the concept. The clue-giver can continue drawing cards to attempt to win the most points within a set time frame. During this time, Team B monitors Team A's clue-giver and drawn card and presses a buzzer provided with the Hasbro game if a forbidden word is used by the clue-giver. If a forbidden word is used, the card is discarded, a new one is drawn, and Team A loses a point. Team B gains a point if Team A draws and then chooses not to play a card. When time is up, any card in play is placed back in the deck, points are counted and recorded, and the opposing team begins its turn. Play continues between the teams until all players have been a clue-giver.^23^

In the PCC, gameplay cards with medical terms and forbidden jargon covered in the previous block were distributed between two teams (Appendix D). One student from the first team described the concept on their card using plain language, as if they were speaking with a patient, while their group attempted to identify the medical concept without seeing the card. Forbidden medical jargon that could not be used when describing the term was listed on the card. For example, a player might have to explain vaccination without using *antibody* or *killed bacteria.* If jargon (either listed on the card or identified by the other team) was used, the other team called out “Jargon!” as the PCC did not use a buzzer. The clue-giver then had one attempt to rephrase using plain language. If they were unable to do so, any member of the other team had the opportunity to attempt a single plain-language description of the concept to win the point. If no participant was able to describe the concept, the card was replaced in the deck. Students could skip cards, and no points were lost for passing on a card, but those cards remained in the deck. One point was awarded for every term correctly identified, and the corresponding card was removed from the deck. Points were tracked by the workshop leader. After 2 minutes, teams switched roles. Within each team, the clue-giver role rotated to all participants. The game concluded and the team with the most points won when all cards had been used or 40 minutes of gameplay had occurred, whichever came first. All participants were clue-givers at least once before the game ended.

The workshop concluded with a 10-minute or longer reflection on jargon avoidance led by the facilitator, which included identifying challenges that had arisen during the game and applying the practiced communication skills to future clinical interactions. As appropriate, the workshop leader used their judgment during the game to arbitrate disagreements, encourage reflection on patient communication among players while discouraging unhealthy competition, and remind players that the goal of the PCC was practice over points. Additionally, because the jargon listed on each concept card was not exhaustive, identification of unlisted jargon terms by players prompted the workshop leader to facilitate discussion while adjusting the time clock as needed. Facilitators prepared ahead of the workshop by reviewing the gameplay instructions PowerPoint (Appendix C) and cards (Appendix D); total preparation time was approximately 30 minutes.

We assessed the impact of 10 workshops from January 2021 to December 2022 through analysis of anonymous evaluations. Postworkshop course evaluation data were collected using a 5-point Likert scale (1 = *strongly disagree,* 5 = *strongly agree*) and free-text responses to provide qualitative feedback via an optional anonymous online survey (Appendix E). This survey was distributed to all participants at the end of every PCC workshop. Responses on jargon avoidance, workshop enjoyment, and workshop recommendation were reported as simple frequencies. Qualitative survey responses were assessed using thematic analysis. Our institutional review board (IRB ID: STUDY00024132, March 2, 2022) found this research did not involve human subjects and therefore did not require further IRB approval.

## Results

Survey responses from eight virtual and two in-person workshops held from January 2021 to December 2022 were assessed. Of the 90 participants during this period, 59% (53 of 90) completed postworkshop surveys. Participation in multiple workshops over the 2 years was possible and did occur but was not recorded due to survey limitations. Demographic data were not collected. Respondents reported both perceived skill gain and enjoyment of the workshop. Of respondents, 91% felt more proficient in avoiding jargon afterwards, and 94% would recommend the workshop (Table 1). All respondents stated they would play again. Common themes were identified from free-text responses (89% response rate, 47 of 53). The number of comments submitted exceeded the number of respondents as participants could submit as many comments as they desired. Positive qualitative feedback was commonly submitted (Table 2). Suggestions for improvement focused on game mechanics (Table 3).

**Table 1.**
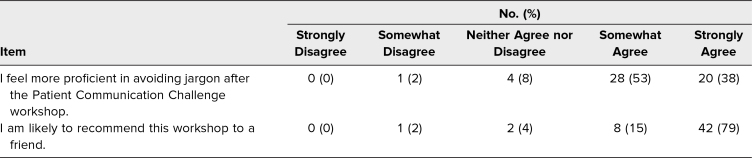
Survey Results for Self-Assessment of Skill Acquisition and Enjoyment (*N* = 53)

**Table 2.**
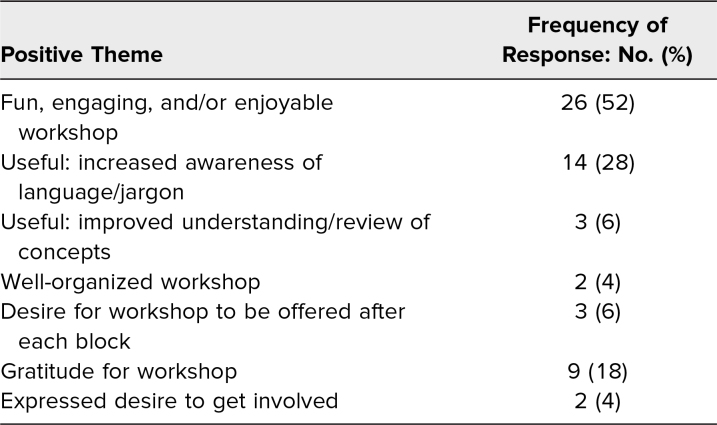
Positive Feedback From Survey Questions 4–7 (*N* = 50)

**Table 3.**
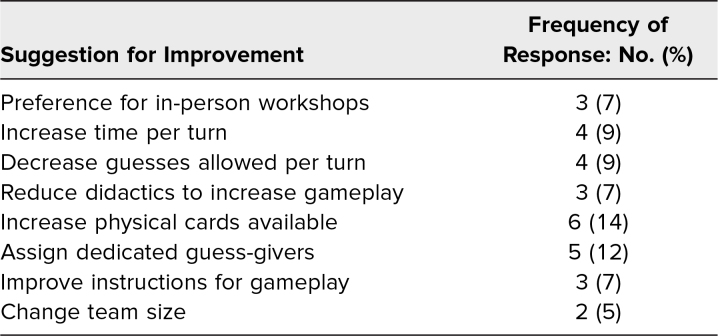
Suggestions for Improvement From Survey (*N* = 43)

## Discussion

Our results indicate that PCC workshops are viable low-cost educational tools for clear communication practice and suggest that gamification is an effective method to practice jargon avoidance. The PCC enables peer-based practice of communication skills transferable to future physicians’ work, which is aligned with experience-based and social learning theories.^28^ Repetition and practice retrieving these concepts also promote retention, understanding, knowledge consolidation, and transfer of this knowledge to different contexts.^26,29^ The workshop is feasible to implement at other institutions given that it requires limited preparation time and inexpensive materials. At our institution, facilitators met after each workshop to review participant feedback and plan iterative changes. Based on these meetings, we made changes to clarify gameplay instructions and time per turn. We recommend that other institutions implement a similar structure for iterative improvement. A barrier to implementation at our institution was the medical students’ busy schedule. We held our workshops during protected postexam time but struggled to expand to other unprotected time, such as evenings, due to poor attendance. We encourage those planning PCC workshops to do so during protected flexible didactic time if possible.

Our workshop expands upon existing health literacy curricula and games by providing a model for frequent, game-based practice of jargon avoidance early in medical training. Our group came across the UK-based jargon-avoidance game^9^ during literature review after independent conceptualization of our workshop. Our workshop is distinct from that commercial game in our focus on medical trainees rather than established practitioners and in our use of American English medical terminology. Nonetheless, our workshop is not without limitations. A game-based workshop has the potential for excessive competition to hinder achievement of the learning objectives. To counteract this potential negative consequence of a gamified activity, workshop facilitators should encourage participants to focus on practicing jargon avoidance over maximizing points.

Regarding limitations in our assessment of the PCC and the workshop, we did not include a pre-exposure assessment, and respondents self-reported their perceived proficiency in avoiding jargon without an objective measurement. Because students self-selected to attend the workshop, they may have had higher baseline clear communication skills or greater motivation to participate in the PCC than other students, possibly biasing the effectiveness of the workshop. Additionally, students could choose to attend one or many workshops, which could not be accounted for in the survey used and thus limited our ability to analyze the PCC's impact on skill development longitudinally. We did not follow up with workshop attendees in the weeks to months after attendance and therefore do not have specific evidence for skill internalization versus attrition over time. However, previous research on medical education suggests that gamification positively impacts learning and knowledge retention.^24^ We theorize that retention and implementation of the communication concepts practiced with the PCC may be greater among those who played the PCC as opposed to those who did not participate in the game.

Future directions for this work include assessment of the PCC's impact on longitudinal skill retention and objective jargon reduction and of its acceptability among non-self-selecting students. Another future direction is expansion of the game to other learners and settings, including those in more advanced stages of medical training, such as clerkship medical students, resident physicians, and faculty in every specialty, as well as trainees in other health professions. Initial efforts at our institution to broaden the use of the PCC to the Schools of Dentistry and Nursing as well as to educators of resident physicians have been favorably received.

The PCC workshops enable medical trainees to practice clear communication skills in a game-based format that adds to a growing body of games focused on health communication. The PCC can be easily implemented by educators and allows for active learning and practice of jargon-free communication by trainees to improve patient care, especially in settings with low health literacy.

## Appendices


PCC Guidelines and Gameplay.docxHealth Literacy Refresher.mp4PCC Workshop Template.pptxPCC Cards.pdfPostworkshop Survey.docx
*All appendices are peer reviewed as integral parts of the Original Publication.*
