# Supplementary material for: A Game-Based Tool for Reducing Jargon Use by Medical Trainees
Source: MedEdPORTAL. 2024 Jun 7;20:11411. doi: 10.15766/mep_2374-8265.11411 (PMC11219083; doi:10.15766/mep_2374-8265.11411)
Supplement: Supplementary file 1 — PCC Guidelines and Gameplay.docxHealth Literacy Refresher.mp4PCC Workshop Template.pptxPCC Cards.pdfPostworkshop Survey.docx [file mep_2374-8265.11411-s001.zip › D. PCC Cards.pdf]

Parasympathetic Nervous System

Peripheral Nervous System

Patient Communication CHALLENGE

Hyperammonemia

Urea cycle
Asterixis
Alpha-ketoglutarate
Glutamine

Patient Communication CHALLENGE

Streptococcus pneumoniae

Gram positive
Lancet-shaped diplococci

Patient Communication CHALLENGE

Systemic Lupus Erythematosus

Autoimmune
Inflammation
Connective tissue
Malar
Nephropathy
Rheumatologic

Patient Communication CHALLENGE

Hypertension

Systole
Diastole
Blood Pressure
Cardiovascular
Vessel
Atherosclerosis

Patient Communication CHALLENGE

Sympathetic Nervous System

Peripheral Nervous System

Patient Communication CHALLENGE

Familial Hypercholesterolemia

LDL receptor
Xanthoma
Lipoprotein

Patient Communication CHALLENGE

NSAID

Cyclooxygenase (COX)
Prostaglandin
Arachidonic acid
Anti-inflammatory
Antipyretic
Analgesic

Patient Communication CHALLENGE

Emphysema

COPD
Alveoli
Pursed lip breathing
Oxygen
Centriacinar/Panacinar
Tripod

Patient Communication CHALLENGE

SIADH

Hormone
Hypothalamus
Pituitary
Vasopressin
Osmolality
Retention

Patient Communication CHALLENGE

Atherosclerosis

Cholesterol
Plaque
Rupture
Macrophages/foam cells

Patient Communication CHALLENGE

Myocardial infarction

Ischemia
Coronary
Circulation
Atherosclerosis
Angina
Occlusion

Patient Communication CHALLENGE

Diabetic Glomerulosclerosis

Basement membrane
Non-enzymatic glycosylation
Hyperfiltration
Microalbuminuria
Glomerulus

Patient Communication CHALLENGE

Urinary Tract Infection

Hematuria
Bacteria
Dysuria
Malodorous
Suprapubic pain

Patient Communication CHALLENGE

Chronic Kidney Disease (CKD)

Glomerulus
Clearance
Filtration
GFR
Proteinuria
Dialysis

Patient Communication CHALLENGE

Diuretic

Hypertension
Hypokalemia
Polyuria
Edema
Nephron (loop of henle, DCT, collecting duct)

Patient Communication CHALLENGE

Pleural Effusion

Exudate
Transudate
CHF
Thoracentesis

Patient Communication CHALLENGE

Nephritic Syndrome

Hematuria
DPGN
RPGN
Crescents

Patient Communication CHALLENGE

Adenocarcinoma

Neoplasm
Peripheral
Columnar Cells
Bronchioloalveolar
Mucin

Patient Communication CHALLENGE

Acute Kidney Injury

BUN
Creatinine
Azotemia
Uremia
Hypovolemic

Patient Communication CHALLENGE

Pneumothorax

Spontaneous
Deviation
Pleural
Hyperresonance
Dyspnea
Fremitus

Patient Communication CHALLENGE

Pulmonary Embolism

Hypercoagulable
DVT
Dyspnea
CT Angiogram
Occlusion

Patient Communication CHALLENGE

Cystic Fibrosis

CFTR gene
Cilia
Chlorine
Bronchiectasis
Trypsinogen

Patient Communication CHALLENGE

Asthma

Atopy
Bronchoconstriction
Prostaglandins/leukotrienes
Beta-receptors
Obstructive
Albuterol

Patient Communication CHALLENGE

Deep Vein Thrombosis

Stasis
Endothelial injury
Hypercoagulable state
Pulmonary emboli
Proximal

Patient Communication
CHALLENGE

Acute Respiratory Distress Syndrome

Alveola
Cytokine
Opacity
Compliance
PEEP
Ventilation

Patient Communication
CHALLENGE

Sarcoidosis

ACE
Autoimmune/rheumatologi
c
Hilar
Non-caseating granuloma
Bell's Palsy
Uveitis

Patient Communication
CHALLENGE

Pneumonia

Lobar
Interstitial/atypical
Congestion
Hepatization
Opacity
Consolidation

Patient Communication
CHALLENGE

Nephrotic Syndrome

Glomerulus
Proteinuria
Hypoalbuminemia
Hyperlipidemia
Podocyte/GBM/mesangial

Patient Communication
CHALLENGE

Pancoast Tumor

Apex
Sympathetic chain
Horner Syndrome
Ptosis/anhidrosis/miosis
Ganglion
SVC syndrome

Patient Communication
CHALLENGE

Pneumoconiosis

Asbestosis/Silicosis/Berylliosis etc.
Calcification
Carcinoma
Fibrosis
Granuloma
Lobe

Patient Communication
CHALLENGE

Pulmonary Hypertension

Arteriosclerosis
Cor Pulmonale
BMPR2
Endothelial
Vasoconstriction

Patient Communication
CHALLENGE

Echocardiogram

Dilation
Stenosis
Cardiomyopathy
Ultrasound
CHF

Patient Communication CHALLENGE

Hypertrophic Cardiomyopathy (HoCM)

Outflow
Myosin
Protein C
Syncope
Systolic/systole
Microfibrillar

Patient Communication CHALLENGE

Aortic Stenosis

Syncope
Angina
Dyspnea
Calcification
Systolic/systole
Murmur

Patient Communication CHALLENGE

Hydronephrosis

Urethral
Pelvis
Calyces
BPH
Obstructive
Creatinine

Patient Communication CHALLENGE

CABG

CAD
Atherosclerosis
Occlusion
Stent
Cardiothoracic

Patient Communication CHALLENGE

ECG

QRS
PR
STEMI/NSTEMI
Inversion
LAD/LCX/RCA/PDA

Patient Communication CHALLENGE

Aneurysm

Prolapse
Stenosis
Dilation
Berry/saccular
Charcot-Bouchard
Lumen

Patient Communication CHALLENGE

Atrial Fibrillation

Arrhythmia
P-wave
Irregularly irregular
Cardioversion
Anticoagulation
Thromboembolic

Patient Communication CHALLENGE

Endocarditis

Sepsis
Embolus
Vegetation

Patient Communication CHALLENGE

Pulmonary Edema

Dyspnea
Hydrostatic pressure
Oncotic pressure
CHF

Patient Communication CHALLENGE

Left sided CHF

Edema
Ejection fraction
Orthopnea
Dyspnea
Diastole/diastolic
Systole/systolic

Patient Communication CHALLENGE

Stent

CAD
Atherosclerosis
Occlusion
CABG

Patient Communication CHALLENGE

Herpes Simplex Virus

Enveloped
Dorsal root ganglia

Patient Communication CHALLENGE

Dialysis

Acidemia
Electrolytes
Uremia
Semipermeable membrane

Patient Communication CHALLENGE

Right sided CHF

Hepatomegaly
JVD
Edema
Cardiomyopathy
Cor pulmonale
Ejection fraction

Patient Communication CHALLENGE

PFT

FEV
FVC
TLC
COPD
Restrictive
Obstructive

Patient Communication CHALLENGE

Lumbar radiculopathy

Nerve root
Compression
Paresthesias
Referred
Sciatica

Patient Communication CHALLENGE

Biopsy

Excision
Incision
Aspiration
Pathology
Specimen

Patient Communication CHALLENGE

Parvovirus B-19

Slapped cheek
Lacy rash
Aplastic anemia
Hydrops fetalis

Patient Communication CHALLENGE

Rheumatoid arthritis

Synovium
Inflammation
Autoimmune
Erythema
MCP/PIP/DIP/MTP
Rheumatologic

Patient Communication CHALLENGE

Rotator Cuff Tendonitis

SITS muscles
Impingement
Abduction
Adduction
Tendinopathy

Patient Communication CHALLENGE

Osteoarthritis

Cartilage
Degenerative
Chronic
Old Age
Crepitus

Patient Communication CHALLENGE

Marfan Syndrome

Genetic defect
Connective tissue
Aortic aneurysm
Lens dislocation
Fibrillin

Patient Communication CHALLENGE

Immunosuppressant

Lymphocytes
Malignancy
Autoimmune
Cytokine
Antibody

Patient Communication CHALLENGE

Pseudogout

Birefringent
Degenerative
Chondrocalcinosis
Calcium pyrophosphate
Osteoarthritis

Patient Communication
CHALLENGE

Osteopetrosis

Resorption
Osteoclast
Cortical
Hematopoiesis
Sclerosis

Patient Communication
CHALLENGE

Bursitis

Patellar
Trochanter
Bursa
Gluteal
Cyst

Patient Communication
CHALLENGE

Carpal Tunnel Syndrome

Entrapment
Median Nerve
Thenar eminence
Paresthesia
Atrophy

Patient Communication
CHALLENGE

Sjogren's Syndrome

Autoimmune
Exocrine
Salivary
Xerostomia
Lymphoma

Patient Communication
CHALLENGE

Avascular Necrosis

Watershed
Infarction
Ischemia
Epiphysis

Patient Communication
CHALLENGE

Compartment Syndrome

Fascia
Outflow obstruction
Necrosis
Anoxia
Hypoxia
Reperfusion

Patient Communication
CHALLENGE

Osteoporosis

Mineralization
Trabecular
Cortical
Estrogen
Vertebral

Patient Communication
CHALLENGE

Raynaud's Phenomenon

Vasospasm
Ischemia
Hypoxia
Reperfusion
SLE
CREST

Patient Communication CHALLENGE

Myasthenia Gravis

Autoimmune
Ptosis
Diplopia
Thymoma
Proximal

Patient Communication CHALLENGE

Ankylosing Spondylitis

Sacroiliac
Uveitis
Costovertebral
Costosternal
Autoimmune

Patient Communication CHALLENGE

Septic arthritis

Synovial
Purulent
Leukocytes
Erythema
Effusion

Patient Communication CHALLENGE

Scleroderma

Sclerodactyly
Pitting
Esophageal dysmotility
Autoimmune
vasculopathy
Fibrosis

Patient Communication CHALLENGE

Lambert-Eaton Syndrome

Autoantibody
Autoimmune
Autonomic
Proximal
Small Cell Lung Cancer

Patient Communication CHALLENGE

Dermatomyositis

Heliotrope
Gottron papules
Erythema
Proximal
CD8
T-cells

Patient Communication CHALLENGE

Enteropathic Arthritis

IBD
Pyoderma Gangrenosun

Patient Communication CHALLENGE

Rhabdomyolysis

Myoglobin
Creatine phosphokinase
Compartment syndrome
AKI

Patient Communication
CHALLENGE

Achondroplasia

Dysplasia
FGFR3
Chondrocytes
Macrocephaly

Patient Communication
CHALLENGE

Stevens-Johnson Syndrome

Bullae
Necrosis
Dermal-epidermal junction
Nikolsky
Erythema Multiforme
TEN

Patient Communication
CHALLENGE

Psoriasis

Acanthosis
Plaque
Auspitz
Arthritis
Autoimmune

Patient Communication
CHALLENGE

Erb palsy

Cervical
Brachial plexus
Suprascapular
Dystocia
"Waiter's tip"

Patient Communication
CHALLENGE

Craniosynostosis

Sutures
Hydrocephalus
Congenital
Fontanelle

Patient Communication
CHALLENGE

Bisphosphonate

Osteoporosis
Hydroxyapatite
Osteoclast
Pyrophosphate

Patient Communication
CHALLENGE

Pemphigus Vulgaris

Bullae
Acantholysis
Desmoglein
Keratinocyte
Antibody
Tombstone

Patient Communication
CHALLENGE

Pityriasis (tinea) versicolor

Malassezia
Potassium hydroxide
"Spaghetti and meatballs"

Patient Communication CHALLENGE

Basal Cell Carcinoma

Basal layer
Epidermis
UV radiation
Tumor suppressor gene

Patient Communication CHALLENGE

Antiphospholipid Syndrome

Thrombosis
Lupus anticoagulant
Anticardiolipin
Antilipoprotein
Hypercoagulable
Autoimmune

Patient Communication CHALLENGE

Osteomalacia

Osteoid
Epiphyseal widening
Osteoblast
Parathyroid
Rickets

Patient Communication CHALLENGE

Pityriasis Rosea

Herald patch
"Christmas tree"
Plaque
Scale

Patient Communication CHALLENGE

Seborrheic Dermatitis

Sebacious glands
Malassezia
Plaque
Scale
Cradle Cap

Patient Communication CHALLENGE

Lichen Planus

Pruritic
Wickham striae
Hypergranulosis
Papule
Plaque
5 P's

Patient Communication CHALLENGE

Gout

Monoarthritis
Urate
Metatarsophalangeal
Tophus
Purines

Patient Communication CHALLENGE

Kawasaki disease

Conjunctival injection
Adenopathy
Strawberry tongue
Erythema
Coronary artery

Patient Communication CHALLENGE

Muscular Dystrophy

Dystrophin gene
Pseudohypertrophy
Gower's sign

Patient Communication CHALLENGE

Guillian-Barre

Ascending paralysis
Demyelination
C. jejuni
Post-infectious

Patient Communication CHALLENGE

Atopic Dermatitis

Hypersensitivity
Pruritus
Flexor
Eczema

Patient Communication CHALLENGE

Meralgia paresthetica

Lateral femoral cutaneous n.
Impingement
Sensory deficits
Inguinal ligament
Superficial

Patient Communication CHALLENGE

Giant cell arteritis

Vascular
Claudication
Corticosteroids
Granuloma
Transmural

Patient Communication CHALLENGE

Radiculopathy

Nerve root
Intervertebral disc
Dermatome
Myotome

Patient Communication CHALLENGE

Impetigo

Staph Aureus
Group A Strep
Vesicles
Bullous

Patient Communication CHALLENGE

Sickle Cell Disease

Hemoglobin
Hemolysis
Point mutation
Asplenia
Vaso-occlusive
Acute chest

Patient Communication CHALLENGE

Rosacea

Vasodilation
Photosensitivity
Erythema

Patient Communication CHALLENGE

Melanoma

Melanocyte
Epidermis
Biopsy
BRAF gene
ABCDE criteria

Patient Communication CHALLENGE

Anterior cruciate ligament tear

Anterior drawer test
Lachman test
Patella
Femoral condyle
Tibia

Patient Communication CHALLENGE

Vancomycin

D-ala-D-ala
MRSA
C. diff

Patient Communication CHALLENGE

MRSA (Methicillin-Resistant Staphylococcus Aureus)

Gram positive
Cocci in clusters

Patient Communication CHALLENGE

Contact dermatitis

T cell
Delayed
Type IV hypersensitivity

Patient Communication CHALLENGE

Celecoxib

Cyclooxygenase (COX)
Gastric
Arachidonic acid
Prostaglandins
Housekeeping

Patient Communication CHALLENGE

Complete Blood Count (CBC)

Lymphocytes
Leukocytes
Erythrocytes
Hemoglobin
Differential

Patient Communication CHALLENGE

Megaloblastic Anemia

Macrocytic
B9/Folate
B12/cyanocobalamin
Hemoglobin
DNA synthesis
Hypersegmented neutrophils

Patient Communication CHALLENGE

Glucocorticoids

Steroid/corticosteroid/corti
sol
Hormone
Adrenal
Immune
system/immunosuppressio
n
Inflammation

Patient Communication CHALLENGE

PCR

Nucleic acid
mRNA
Reverse transcriptase
Amplify
Complementary
Denaturation

Patient Communication CHALLENGE

Leukopenia

Granulocytes
Chemotherapy
Immunosuppression

Patient Communication CHALLENGE

Thalassemia

Hemoglobin
Mutation
Deletion
Microcytic anemia
Transfusion

Patient Communication CHALLENGE

Anemia

Hemoglobin
Hematocrit
Reticulocyte

Patient Communication CHALLENGE

Vaccination

Immunization
Attenuated
Inactivated
Inoculate
mRNA

Patient Communication CHALLENGE

Lymphadenopathy

Reticuloendothelial system
Lymph nodes

Patient Communication CHALLENGE

Complement System

Opsonization
Anaphylaxis
Membrane attack complex (MAC)

Patient Communication CHALLENGE

Cluster of Differentiation (CD) Markers

Flow cytometry
Immunotherapy

Patient Communication CHALLENGE

Polycythemia

EPO
Plethora
Hemoglobin
Hematocrit

Patient Communication CHALLENGE

Varicella Zoster

Chicken Pox
Shingles
Herpes
Dermatome
Dorsal Root Ganglion

Patient Communication CHALLENGE

Paroxysmal Nocturnal Hemoglobinuria (PNH)

Complement
MAC
Hemolysis
Eculizumab

Patient Communication CHALLENGE

Adaptive Immunity

Humoral
Cell-mediated
B lymphocyte (B-cell)
T-cell

Patient Communication CHALLENGE

Neutropenia

Granulocytes
Chemotherapy
Immunosuppression

Patient Communication CHALLENGE

Von Willebrand Disease

Factor VIII
Von Willebrand Factor
Desmopressin

Patient Communication CHALLENGE

Thrombolysis

Fibrinogen
Fibrin
D-dimer

Patient Communication CHALLENGE

PTT

Intrinsic
Clotting factors

Patient Communication CHALLENGE

Type I Hypersensitivity

Mast cell degranulation
Histamine
IgE
Epinephrine

Patient Communication CHALLENGE

Hemophilia

Factor VIII
Factor IX
Desmopressin
PTT
Emicizumab

Patient Communication CHALLENGE

Thrombotic Thrombocytopenic Purpura

ADAMTS13
Von Willebrand Factor
Schistocytes

Patient Communication CHALLENGE

PT/INR

Extrinsic
Clotting factors
Warfarin/Coumadin

Patient Communication CHALLENGE

Type IV Hypersensitivity

Dermatitis
Memory T Cells (CD4+)
Cytotoxic T Cells (CD8+)
Dermatitis

Patient Communication CHALLENGE

Plasma

Clotting Factors
INR
Frozen

Patient Communication
CHALLENGE

CMV

Owl eye
Herpes
Congenital

Patient Communication
CHALLENGE

Asplenia

Encapsulated Bacteria
lymphoid organ
Sickle Cell

Patient Communication
CHALLENGE

Warfarin

Bridge
Heprin

Patient Communication
CHALLENGE

Blood Typing

Antibody
Antigen
Type A, Type B, Type O
Rh

Patient Communication
CHALLENGE

DiGeorge

22q11 deletion
Velocardiofacial
CATCH-22

Patient Communication
CHALLENGE

Malaria

Trophozoites
Babesia

Patient Communication
CHALLENGE

Heparin

Anticoagulation
Warfarin

Patient Communication
CHALLENGE

Autoimmune Disease

Antibody
Immunosuppression
T/B-cell
IgG/IgM/IgA

Patient Communication
CHALLENGE

Antigen test

Immunoassay
Enzyme
Substrate
Antibody
Blot
ELISA

Patient Communication
CHALLENGE

HIV

IV drug user
AIDS
CD4 count

Patient Communication
CHALLENGE

Massive Transfusion Protocol

Type and screen

Patient Communication
CHALLENGE

Leukemia

Lympho-
Myelo-
B/T-cell
Neoplasm
Malignant
Hematopoietic

Patient Communication
CHALLENGE

Hypercoagulable

Thrombosis/DVT
Mutation
Factor
PT/PTT

Patient Communication
CHALLENGE

Factor V Leiden

Hypercoagulable
Coagulation cascade

Patient Communication
CHALLENGE

Bone Marrow Transplant

Graft versus Host

Patient Communication
CHALLENGE

Osteogenesis Imperfecta

Sclera
Fracture
Collagen
Triple helix

Patient Communication CHALLENGE

Autosomal Recessive

Allele
Chromosome
Carrier

Patient Communication CHALLENGE

Antibiotics

Bacteria
Lysis
Gram Negative/Positive
Resistance

Patient Communication CHALLENGE

Chemotherapy

Cytotoxic
Apoptosis
Mitosis
Myelosuppression
Alopecia

Patient Communication CHALLENGE

Li Fraumeni Syndrome

Tumor suppressor
Cell cycle
p53

Patient Communication CHALLENGE

G6PD Deficiency

Glutathione
Oxidative Stress
Free radical
Hemolysis

Patient Communication CHALLENGE

Xeroderma Pigmentosum

Genetic defect
DNA repair
Enzyme
Hyperpigmentation
Nucleotide excision repair

Patient Communication CHALLENGE

Heart Murmer

Auscultate
Valves
Rub
Gallop

Patient Communication CHALLENGE

Sensitivity (of a test)

True positive
False negative
Probability
Gold standard

Patient Communication CHALLENGE

Botulism

Toxin
Flaccid paralysis
Floppy baby
SNARE protein
Neuromuscular junction

Patient Communication CHALLENGE

CYP450 Inducer

Metabolism
Therapeutic level
Bioavailability
Enzyme

Patient Communication CHALLENGE

Down Syndrome

Trisomy
Nondisjunction
Meiosis
Advanced maternal age

Patient Communication CHALLENGE

Bioavailability

First pass metabolism
Therapeutic level
Concentration

Patient Communication CHALLENGE

Scurvy

Deficiency
Hydroxylation
Petechiae
Gingival edema
Cross-link

Patient Communication CHALLENGE

Clostridium Difficile Infection

Gram positive
Anaerobic
Spore forming
Iatrogenic
Oral vancomycin

Patient Communication CHALLENGE

Sacrococcygeal Teratoma

Primitive streak
Coccyx
Benign

Patient Communication CHALLENGE

Tetanus

Gram positive
Spastic paralysis
SNARE
Spore
Neuromuscular junction

Patient Communication CHALLENGE

Von Gierke Disease

Glycogen
Glucose
Glucose-6-phosphatase
Hypoglycemia

Patient Communication CHALLENGE

Neural Tube Defect

Folate
Folic acid
Anencephaly
Spina bifida
Neuropore

Patient Communication CHALLENGE

Huntington's Disease

CAG
Anticipation
Trinucleotide repeat
Chorea
Autosomal dominant

Patient Communication CHALLENGE

Anticipation

Trinucleotide repeat
Huntington's

Patient Communication CHALLENGE

Lumbar Puncture

Subarachnoid space
L3/L4
Dura
Arachnoid
Meninges
Spinal tap

Patient Communication CHALLENGE

Teratogen

Embryo
Exposure
Gestation
Organogenesis

Patient Communication CHALLENGE

Prader Willi Syndrome

Paternal deletion
Silencing
Chromosome 15
Obesity
Imprinting

Patient Communication CHALLENGE

Spina Bifida

Caudal neuropore
Neural
Folic acid
Vertebral arch

Patient Communication CHALLENGE

MRI

CT-scan
X-ray

Patient Communication CHALLENGE

Lysosomal Storage Disease

Sphingolipidoses
Metabolism

Patient Communication CHALLENGE

Phenylketonuria (PKU)

Phenylalanine hydroxylase
Tetrahydrobiopterin (BH4)
Tyrosine

Patient Communication CHALLENGE

Mitochondrial Inheritance

Mutation
Powerhouse
Heteroplasmy

Patient Communication CHALLENGE

Diabetic Ketoacidosis

Noncompliance
Ketosis
Kussmaul respiration
Metabolic acidosis

Patient Communication CHALLENGE

Specificity (of a test)

True negative
False positive
Probability

Patient Communication CHALLENGE

Glycogen Storage Disease

Metabolism

Patient Communication CHALLENGE

Mosacism

Allele
Chromosome
Tortise shell cat

Patient Communication CHALLENGE

Variable Expressivity

Allele
Chromosome
Phenotype

Patient Communication CHALLENGE

Cru-di-chat

Cat
Microdeletion
Congenital

Patient Communication CHALLENGE

Wolf-Hirschhorn

Greek Helmet
Microdeletion
Congenital

Patient Communication CHALLENGE

X-Linked Disease

Allele
Chromosome
Gene

Patient Communication CHALLENGE

P-Value

Significant
< 0.05

Patient Communication CHALLENGE

Chediak-Higashi

Albinism
Reccurent pyrogenic
LYST gene

Patient Communication CHALLENGE

Williams Syndrome

Cocktail personality
Elfin face
Congenital

Patient Communication CHALLENGE

Ulcerative Colitis

TH2
Mucosal/Submucosal
Crypt
Haustra
Sclerosing cholangitis
Lead pipe

Patient Communication CHALLENGE

Shock

Hypotensive
Septic
Distributive
Cardiogenic
Anaphylactic

Patient Communication CHALLENGE

Diabetes

Glucose
Insulin
Resistance
Beta Cells
Autoimmune

Patient Communication CHALLENGE

Lesch Nyhan

Gout
Hyperuricemia
Purine
HGPRT

Patient Communication CHALLENGE

Diverticulosis

Intraluminal
Villi
False diverticula

Patient Communication CHALLENGE

Crohn's Disease

Cobblestone
Granuloma
TH1
Erythema Nodosum
Pyoderma Gangrenosum
Spondyloarthritis

Patient Communication CHALLENGE

Coronary Artery Disease

Atherosclerosis
Embolus/Emboli/Embolism
Thrombi/Thrombus
Ischemia

Patient Communication CHALLENGE

Chronic Granulomatous Disease

NADPH Oxidase
Respiratory Burst
Catalase Positive
Non-Caseating Granulomas

Patient Communication CHALLENGE

Acute Gastritis

NSAID
Curling ulcer
Mucosal ischemia
Cushing ulcer
Vagal stimulation

Patient Communication CHALLENGE

Plummer-Vinson Syndrome

Dysphagia
Anemia
Esophageal web
Glossitis
SCC

Patient Communication CHALLENGE

Achalasia

LES
Myenteric plexus
Stenosis
Chagas disease

Patient Communication CHALLENGE

Diverticulitis

LLQ
Leukocytosis
Abscess
Fistula
Peritonitis

Patient Communication CHALLENGE

Zenker Diverticulum

False diverticulum
Esophageal dysmotility
Herniation
Pharyngeal
Dysphagia
Aspiration

Patient Communication CHALLENGE

Barrett's Esophagus

Metaplasia
Stratified squamous epithelium
Columnar cell
Goblet cell
GERD
Adenocarcinoma

Patient Communication CHALLENGE

Mallory-Weiss Syndrome

Longitudinal laceration
GE junction
Mucosa/submucosa
Hematemesis
Bulimia

Patient Communication CHALLENGE

Appendicitis

Lymphoid hyperplasia
Closed-loop
Intraluminal
Peritonitis
Ectopic pregnancy

Patient Communication CHALLENGE

Portal Hypertension

Cirrhosis
Vascular obstruction
Thrombosis
Schistosomiasis
Varices
Hematemesis

Patient Communication CHALLENGE

Polyp

Sessile
Pedunculated
Protrusion
Lumen
Adenomatous
Serrated

Patient Communication CHALLENGE

Volvulus

Mesentery
Infarction
Obstruction
Midgut
Sigmoid

Patient Communication CHALLENGE

Meckel Diverticulum

True diverticulum
Vitelline duct
Ectopic acid
Hematochezia
Volvulus
Intussusception

Patient Communication CHALLENGE

GERD

H. pylori
Epigastric
LES

Patient Communication CHALLENGE

Cirrhosis

Fibrosis
Hepatitis
Stellate cells
Biliary
Nodule

Patient Communication CHALLENGE

Ileus

Hypomotility
Flatus
Distension
Opiate
Hypokalemia

Patient Communication CHALLENGE

Hirschsprung Disease

Megacolon
Ganglion cells
Enteric plexus
Auerbach/Meissner
Neural crest cells
Down Syndrome

Patient Communication CHALLENGE

Endoscopy

Esophagogastroduodenoscopy (EGD)

Patient Communication CHALLENGE

ERCP (Endoscopic Retrograde Cholangiopancreatography)

Endoscopy
Fluoroscopy
Cholelithiasis

Patient Communication CHALLENGE

Cholecystitis

Calculous
Acalculous
HIDA scan
Murphy's sign

Patient Communication CHALLENGE

Pyloric Stenosis

Sphincter
Epigastric

Patient Communication CHALLENGE

Celiac Disease

Tissue transglutaminase (TTG)
Villous atrophy
Crypt hyperplasia
Lymphocytosis
Gliadin

Patient Communication CHALLENGE

Proton Pump Inhibitor

Parietal cells
Na+/H+ ATPase
Zollinger-Ellison Syndrome

Patient Communication CHALLENGE

Biliary Colic

Neurohormonal activation
Cholelithiasis
RUQ

Patient Communication CHALLENGE

Pancreatitis

Autodigestion
Epigastric
Amylase
Lipase
Necrosis

Patient Communication CHALLENGE

Liver Panel

Alanine transaminase (ALT)
Aspartate transaminase (AST)
Alkaline phosphatase (ALP)
Albumin
Gamma Glutamyl transferase (GGT)

Patient Communication CHALLENGE

Peptic Ulcer Disease

Mucosal protection
Zollinger-Ellison Syndrome

Patient Communication CHALLENGE

Jaundice

Bilirubin
Sclera
Conjugated

Patient Communication CHALLENGE

Fistula

Transmural
Ostomy

Patient Communication CHALLENGE

Prolactinoma

Amenorrhea
Bitemporal hemianopsia
Transsphenoidal

Patient Communication CHALLENGE

Lynch Syndrome

Mismatch repair
Hereditary

Patient Communication CHALLENGE

Bilirubin

Hemolysis
Cirrhosis

Patient Communication CHALLENGE

Hernia

Inguinal
Umbilical
Fascia

Patient Communication CHALLENGE

Pheochromocytoma

Medulla
Chromaffin Cells
Catecholamines
Polycythemia

Patient Communication CHALLENGE

Hashimoto Thyroiditis

Antithyroglobulin
Antithyroid peroxidase
Thyrotoxicosis
Hurthle cells

Patient Communication CHALLENGE

Cushing Syndrome

Corticosteroids
Adrenal adenoma
Striae

Patient Communication CHALLENGE

Acromegaly

Pituitary adenoma
IGF-1

Patient Communication CHALLENGE

Diabetes Insipidus

Antidiuretic hormone
Polyuria
Polydipsia
Nephrogenic

Patient Communication CHALLENGE

Adrenal Insufficiency

Glucocorticoids
Mineralocorticoids
Orthostasis

Patient Communication CHALLENGE

Graves Disease

Immunoglobulin
Hypersensitivity
Exophthalmos

Patient Communication CHALLENGE

SIADH

Hyponatremia
Osmolality
Osmotic demyelination

Patient Communication CHALLENGE

Hemochromatosis

HFE gene
Hemosiderin
Cardiomyopathy
Ferritin

Patient Communication CHALLENGE

Irritable Bowel Syndrome

Defecation
Structural abnormalities

Patient Communication CHALLENGE

Gilbert's Disease

UDP glucuronosyltransferase
Unconjugated bilirubin

Patient Communication CHALLENGE

Sheehan Syndrome

Hypopituitarism
Hypoperfusion
Hemorrhage
Amenorrhea

Patient Communication CHALLENGE

Cholelithiasis

Bile salts
Pigmented
Radiolucent

Patient Communication CHALLENGE

Physiologic Neonatal Jaundice

UDP glucuronosyltransferase
Hyperbilirubinemia
Neonate

Patient Communication CHALLENGE

Hyperparathyroidism

Hypercalcemia
Hypercalciuria
Hypophosphatemia
Adenoma
Osteitis fibrosa cystica

Patient Communication CHALLENGE

Hyperosmolar Hyperglycemic State (HHS)

Osmolality
Diuresis
Polyuria

Patient Communication CHALLENGE

Parkinson Disease

Lewy bodies
Tremor

Patient Communication CHALLENGE

Delirium

Reversible
Dementia

Patient Communication CHALLENGE

Spongiform Encephalopathy

Creutzfeldt-Jakob
Prions
Mad-cow disease

Patient Communication CHALLENGE

Thyroid Storm

Tachyarrhythmia

Patient Communication CHALLENGE

Meningitis

Meningismus
S. pneumo, etc
Nuchal rigidity

Patient Communication CHALLENGE

Huntington Disease

Huntingtin gene (HTT)
Chorea
CAG

Patient Communication CHALLENGE

Alzheimer Dementia

Neurofibrillary tangles
Beta-amyloid
Insidious
Senile plaques

Patient Communication CHALLENGE

MEN (Multiple Endocrine Neoplasms)

Adenoma
Hyperplasia
RET
MEN1

Patient Communication CHALLENGE

Aphasia

Wernicke
Broca
Receptive
Expressive

Patient Communication CHALLENGE

Spinal Muscular Atrophy

Floppy baby
Fasciculations
SMN1
Werdnig-hoffman

Patient Communication CHALLENGE

Absence Seizure

Hyperventilation
Petit mal
Postictal

Patient Communication CHALLENGE

Seizures

Epilepsy
Grand mal/tonic-clonic
Postictal
Febrile

Patient Communication CHALLENGE

OCD

Ego-dystonic

Patient Communication CHALLENGE

Mania

Bipolar
Grandiosity
Depression

Patient Communication CHALLENGE

Multiple Sclerosis

Optic neuritis
Lhermitte's
CNS
Axonal damage
Oligoclonal bands

Patient Communication CHALLENGE

Meningioma

Arachnoid cells
Extra-axial
Meninges

Patient Communication CHALLENGE

Major Depressive Disorder

Depressed
Anhedonia

Patient Communication CHALLENGE

Narcolepsy

Orexin
Hypocretin
Lateral hypothalamus
Cataplexy

Patient Communication CHALLENGE

Hydrocephalus

Intracranial pressure (ICP)
Communicating
Non-communicating
Cerebrospinal fluid

Patient Communication CHALLENGE

Stroke

Infarction
Occlusion
tPA
Hemorrhagic
Hemiplegia
Hemiparesis

Patient Communication CHALLENGE

ADHD

Hyper
Fidgeting
Stimulants

Patient Communication CHALLENGE

PTSD

Veteran
Flashbacks

Patient Communication CHALLENGE

Schizophrenia

Delusions
Hallucinations
Word salad

Patient Communication CHALLENGE

Demyelinating

Schwann Cell
Oligodendrocyte
Multiple Sclerosis
Guillain-Barre

Patient Communication CHALLENGE

Amyotrophic Lateral Sclerosis

Lou Gehrig disease
UMN + LMN
Superoxide dismutase
ALS

Migraine

Photophobia
Phonophobia
Meninges

Cluster Headache

Periorbital
Lacrimation
Rhinorrhea
Trigeminal

Transient Ischemic Attack (TIA)

Mini-stroke
Ischemia

Patient Communication CHALLENGE
Patient Communication CHALLENGE
Patient Communication CHALLENGE
Patient Communication CHALLENGE

Bell's Palsy

Idiopathic
Lyme

Extrapyramidal Symptoms

Dystonia
Akathisia
Parkinsonism
Tardive dyskinesia
Antipsychotics

Aneurysm

Berry
Saccular
Dilation

Subdural Hematoma

Crescent-shaped
Hyperdense
Bridging veins

Patient Communication CHALLENGE
Patient Communication CHALLENGE
Patient Communication CHALLENGE
Patient Communication CHALLENGE

Locked-in Syndrome

Basilar artery
Corticospinal
Corticobulbar

Patient Communication CHALLENGE

Epidural Hematoma

Middle meningeal
Lucid interval
Biconvex

Patient Communication CHALLENGE

Circadian Rhythm

Suprachiasmatic nucleus
Melatonin
Entrain

Patient Communication CHALLENGE

Conjunctivitis

Conjunctiva
Adenovirus

Patient Communication CHALLENGE

Wernicke (Receptive) Aphasia

Temporal
Fluent

Patient Communication CHALLENGE

Subarachnoid Hemorrhage

Aneurysm
AVM
Xanthochromic

Patient Communication CHALLENGE

Ataxia

Cerebellar
Trendelenburg

Patient Communication CHALLENGE

Syringomyelia

Central cord syndrome
Syrinx
Chiari malformation

Patient Communication CHALLENGE

Factitious Disorder

Munchausen
Gain

Patient Communication CHALLENGE

Benign Paroxysmal Peripheral Vertigo

Otolith
Dix-Hallpike
Epley

Patient Communication CHALLENGE

Asterixis

Hepatic encephalopathy
Wilson disease

Patient Communication CHALLENGE

Broca (Expressive) Aphasia

Frontal gyrus
Nonfluent

Patient Communication CHALLENGE

Neuroleptic Malignant Syndrome

Antipsychotics
Myoglobinuria
Encephalopathy

Patient Communication CHALLENGE

Horner Syndrome

Ptosis
Miosis
Anhidrosis
Pancoast tumor

Patient Communication CHALLENGE

Osmotic Demyelination

Hyponatremia
Pontine myelinolysis
Osmotic
Iatrogenic

Patient Communication CHALLENGE

Chorea

Basal ganglia
Huntington
Rheumatic fever

Patient Communication CHALLENGE

Patient Communication
CHALLENGE

Platelets

Megakaryocytes
Thrombopoietin
Von Willebrand Factor

Patient Communication
CHALLENGE

McArdle Disease

Enzyme
Lysosome

Patient Communication
CHALLENGE

Delirium Tremens

Autonomic
Psychomotor agitation
Gamma-Glutamyl
transferase (GGT)

Patient Communication
CHALLENGE

Patient Communication
CHALLENGE

Patient Communication
CHALLENGE

T-cell Activation

MHC
Co-Stimulation
Combinatorial
Junctional
AIRE

Patient Communication
CHALLENGE

Leukocyte Adhesion Deficiency

Umbilical cord
Macrophage
Immunodifiency
reccurent infection

Patient Communication
CHALLENGE
