# Supplementary material for: A Game-Based Tool for Reducing Jargon Use by Medical Trainees
Source: MedEdPORTAL. 2024 Jun 7;20:11411. doi: 10.15766/mep_2374-8265.11411 (PMC11219083; doi:10.15766/mep_2374-8265.11411)
Supplement: Supplementary file 1 — PCC Guidelines and Gameplay.docxHealth Literacy Refresher.mp4PCC Workshop Template.pptxPCC Cards.pdfPostworkshop Survey.docx [file mep_2374-8265.11411-s001.zip › E. Postworkshop Survey.docx]

Post-Workshop Survey:

1. After the workshop, I feel more proficient in avoiding jargon?

1: Strongly disagree

2: Somewhat disagree

3: Neither agree nor disagree

4: Somewhat agree

5: Strongly agree

1. If this game were readily available, I would play it again.

1: Strongly disagree

2: Somewhat disagree

3: Neither agree nor disagree

4: Somewhat agree

5: Strongly agree

1. I am likely to recommend this workshop to a friend.

1: Strongly disagree

2: Somewhat disagree

3: Neither agree nor disagree

4: Somewhat agree

5: Strongly agree

1. What did you enjoy about the medical taboo game?
2. How could the game run more smoothly?
3. Did 2 minutes per turn feel too long/short/just right?
4. Any other comments/suggestions?
5. Please include your email if you'd like us to keep in touch.
