# Supplementary material for: A Game-Based Tool for Reducing Jargon Use by Medical Trainees
Source: MedEdPORTAL. 2024 Jun 7;20:11411. doi: 10.15766/mep_2374-8265.11411 (PMC11219083; doi:10.15766/mep_2374-8265.11411)
Supplement: Supplementary file 1 — PCC Guidelines and Gameplay.docxHealth Literacy Refresher.mp4PCC Workshop Template.pptxPCC Cards.pdfPostworkshop Survey.docx [file mep_2374-8265.11411-s001.zip › A. PCC Guidelines and Gameplay.docx]

The Patient Communication Challenge: Guidelines and Gameplay

Thank you for choosing the Patient Communication Challenge (PCC) to improve patient-centered communication skills! The PCC requires a small amount of preparation before the workshop. We encourage you to follow the guidelines and timeline below, and to adapt the structure and materials as needed for your learning environment and goals. Have fun and remember: “practice over points”!

Table of Contents:

[Workshop Timeline: 2](#_Toc156563761)

[One Week Prior to the Workshop: 2](#_Toc156563762)

[On the Day of the Workshop: 2](#_Toc156563763)

[Equipment Needed 3](#_Toc156563764)

[Gameplay Instructions 3](#_Toc156563765)

[Medical Jargon 4](#_Toc156563766)

[Skipping Cards 4](#_Toc156563767)

[Scoring 4](#_Toc156563768)

[Best Practices 4](#_Toc156563769)

[References 4](#_Toc156563770)

# Workshop Timeline:

## One Week Prior to the Workshop:

| Task | Comments | Expected Duration |
| --- | --- | --- |
| Send out the Health Literacy Refresher video (Appendix B) via email to all participants | This video is a brief review of major health literacy concepts taught at the authors’ institution to be watched by participants prior to playing the PCC. It is not meant to be stand-alone curriculum on health literacy. The authors encourage personalizing health literacy education to the audience and context and developing a health literacy and clear communication curriculum if this is lacking at an organization or institution.  Additional information on best practices for clear communication can be found in the referenced literature.^1-3^ | 10 minutes |
| Prepare the Patient Communication Challenge concept cards | Sort these cards to include the topics appropriate for your audience. The authors recommend the cards be printed and cut out with a large paper cutter before the workshop. If the cards will be used in multiple workshops, printing on thicker card stock paper and laminating the cards is recommended to improve their longevity. | 30 minutes |
| Review Gameplay Instructions | Review the Patient Communication Challenge gameplay instructions in this document and the PowerPoint presentation *Template for PCC Workshop* (Appendix C) to ensure familiarity with the game. | As needed |

## On the Day of the Workshop:

| Task | Comments | Expected Duration |
| --- | --- | --- |
| Welcome the participants and introduce the Patient Communication Challenge | Utilize the PowerPoint presentation *Template for PCC Workshop* to review objectives, gameplay, and display rules during the game as needed (Appendix C). | 10 minutes |
| Play the Patient Communication Challenge | Facilitators prepare ahead of the workshop by reviewing the gameplay instructions (in this document and Appendix C) and the gameplay cards (Appendix D).  The workshop leader may need to use their judgement during the game to arbitrate disagreements, to encourage reflection on patient communication among players while discouraging unhealthy competition, and to remind players the goal is “practice over points”.  Facilitators should note the jargon listed on each concept card is not an exhaustive list of all possible medical jargon. Identification of unlisted jargon terms by players should prompt the workshop leader to facilitate discussion while adjusting the timeclock as needed. | 40 minutes |
| Group Debrief and Reflection | Topics for reflection may include, but are not limited to:   - Challenges encountered and lessons learned during the game - How the practiced communication skills can be applied to future clinical interactions - Potential barriers to the use of plain language | 10 minutes |
| Distribute the Post-Workshop Survey | The Post-Workshop Survey (Appendix E) may be distributed to participants in person or via email. Facilitators are encouraged to adapt the survey if desired for their goals and needs. | 10 minutes |

# Equipment Needed

To play the Patient Communication Challenge, you will need:

- The Health Literacy Refresher Video^4^ (Appendix B)
- The Patient Communication Challenge PowerPoint presentation (Appendix C) and the ability and equipment to present it
- The Patient Communication Challenge concept cards^5^ (Appendix D)
- A method to record points such as a dry erase board
- A timekeeping device
- The Post-Workshop Survey (Appendix E)

# Gameplay Instructions

1. Split participants into two equal teams (Team One and Team Two) and identify the first clue-giver on each team.
2. Divide gameplay concept cards with medical terms and forbidden jargon covered in the previous block between the two teams, placing the cards face-down in the center of each team.
3. Team One’s first clue-giver draws a card and describes the concept on the card to their teammates using plain language, as if they were speaking with a patient, without using the forbidden medical jargon listed on the card.
4. The other members of Team One shout out their guesses and the clue-giver identifies the correct answer when it is guessed. The guessed card is placed face-up in a separate pile and the clue-giver continues pulling and describing cards until two minutes have passed.
5. The facilitator counts the number of correctly guessed cards and records one point per card. These cards are then discarded.
6. Team Two’s first clue-giver begins their turn, repeating steps 3 through 5, until two minutes have passed. At this point, Team One’s second clue-giver begins their turn. The teams go back and forth, rotating through clue-givers, until all cards are used or the allotted time for gameplay in the workshop has passed.

## Medical Jargon

- While one team is playing, the other should be listening for the use of medical jargon during description of a concept. If jargon is heard, any member of the listening team should call “jargon!”, at which point gameplay should stop and the member that called out jargon identifies what they heard.
- The clue-giver has one attempt to rephrase using plain language. If they are unable to do so, any member of the listening team may attempt a single plain language description of the concept to win the point.
- If no participant is able to describe the concept using plain language, the card is replaced into the deck. After the point is won or lost, the clue-giver continues with their turn for the time remaining.

## Skipping Cards

- Participants may skip cards. There are no points lost for passing on a card, but these cards remain in the deck.

## Scoring

- One point is awarded for every term correctly identified and that card is removed from the deck. There is no way to lose points.
- Points are tracked on a whiteboard by the workshop leader.
- The winning team is the team with the most points at the end of the workshop.

## Best Practices

- The goal of the PCC is “practice over points”, which means prioritizing practicing communicating in ways future patients will understand over winning the game.
- The workshop leader may need to use their judgement during the game to arbitrate disagreements, to encourage reflection on patient communication among players while discouraging unhealthy competition, and to remind players the goal of the PCC is “practice over points”.
- Facilitators should note that the jargon listed on each concept card is not an exhaustive list, so identification of unlisted jargon terms by players should prompt the workshop leader to facilitate discussion while adjusting the timeclock as needed.

# References

1. DeWalt DA, Callahan LF, Hawk VH, et al. Health Literacy Universal Precautions Toolkit. Agency for Healthcare Research and Quality. <https://www.ahrq.gov/sites/default/files/wysiwyg/professionals/quality-patient-safety/quality-resources/tools/literacy-toolkit/healthliteracytoolkit.pdf>

2. Coleman C, Salcido-Torres F, Cantone RE. "What Questions Do You Have?": Teaching Medical Students to Use an Open-Ended Phrase for Eliciting Patients' Questions. *Health Lit Res Pract*. Jan 2022;6(1):e12-e16. doi:10.3928/24748307-20211206-01

3. Coleman CA, Peterson-Perry S, Bumsted T. Long-Term Effects of a Health Literacy Curriculum for Medical Students. *Fam Med*. Jan 2016;48(1):49-53.

4. Herzig M, Burney E. Health Literacy Refresher. Video. 2024. <https://youtu.be/aT4_FkZLxfY>

5. Burney E, Arora M, Gaillard M, Herzig M, Lester L, Park S. Patient communication challenge game concept cards. Oregon Health & Science University; 2023.
