# Supplementary material for: A Game-Based Tool for Reducing Jargon Use by Medical Trainees
Source: MedEdPORTAL. 2024 Jun 7;20:11411. doi: 10.15766/mep_2374-8265.11411 (PMC11219083; doi:10.15766/mep_2374-8265.11411)
Supplement: Supplementary file 1 — PCC Guidelines and Gameplay.docxHealth Literacy Refresher.mp4PCC Workshop Template.pptxPCC Cards.pdfPostworkshop Survey.docx [file mep_2374-8265.11411-s001.zip › C. PCC Workshop Template.pptx]

## Slide 1
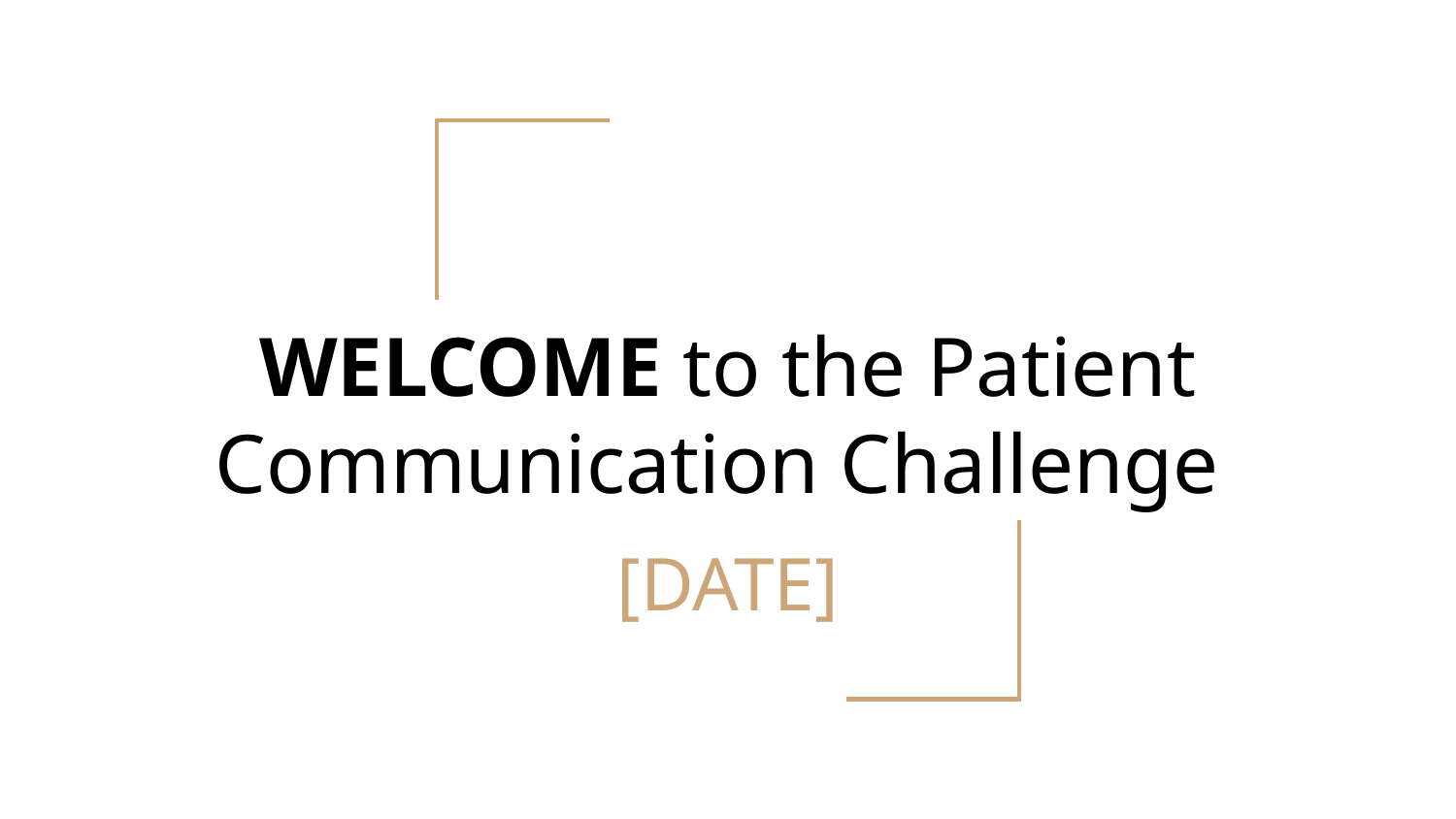

# WELCOME to the Patient Communication Challenge
[DATE]

## Slide 2
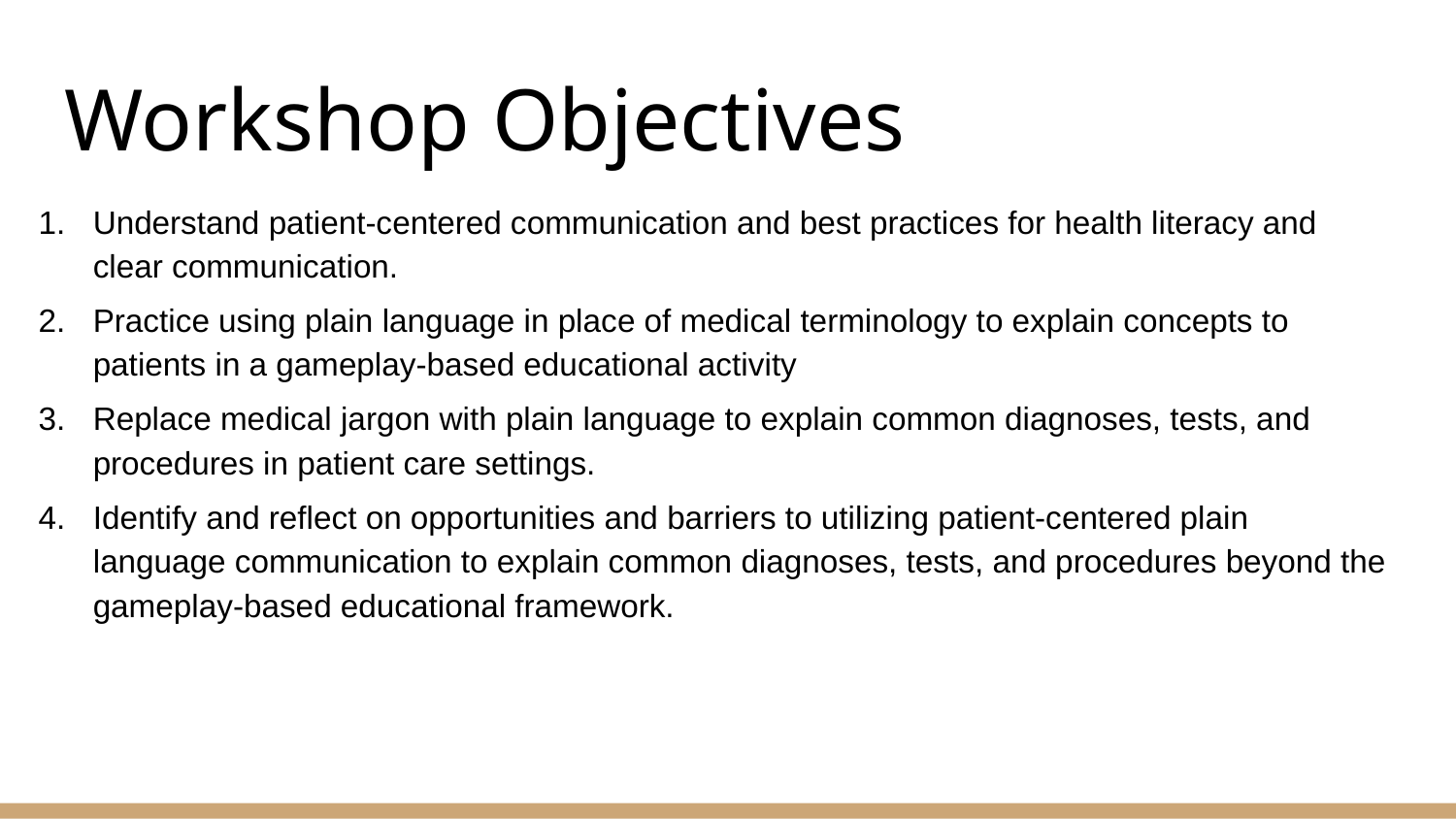

# Workshop Objectives
Understand patient-centered communication and best practices for health literacy and clear communication.
Practice using plain language in place of medical terminology to explain concepts to patients in a gameplay-based educational activity
Replace medical jargon with plain language to explain common diagnoses, tests, and procedures in patient care settings.
Identify and reflect on opportunities and barriers to utilizing patient-centered plain language communication to explain common diagnoses, tests, and procedures beyond the gameplay-based educational framework.

## Slide 3
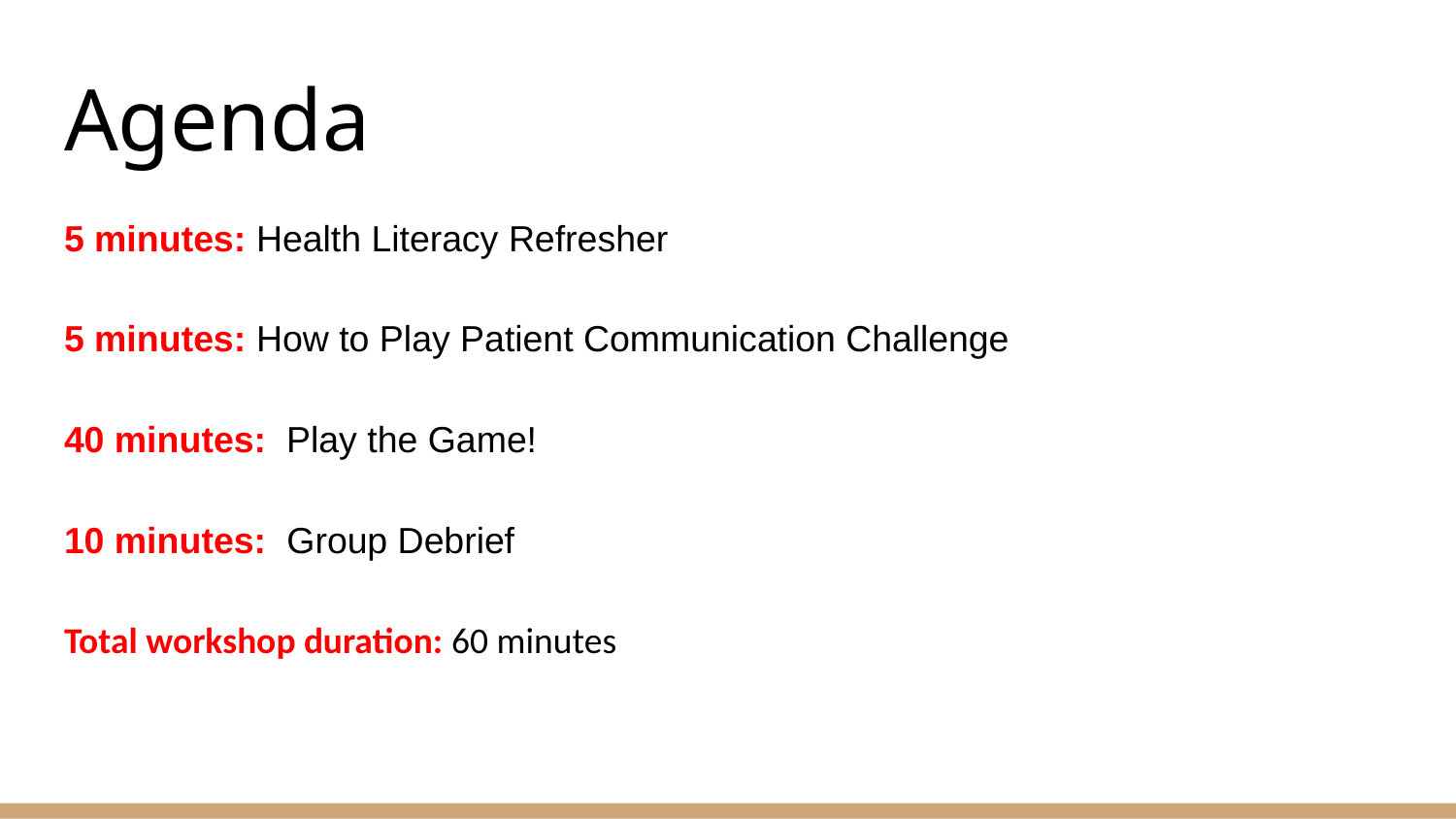

# Agenda
5 minutes: Health Literacy Refresher
5 minutes: How to Play Patient Communication Challenge
40 minutes:  Play the Game!
10 minutes:  Group Debrief
Total workshop duration: 60 minutes

## Slide 4
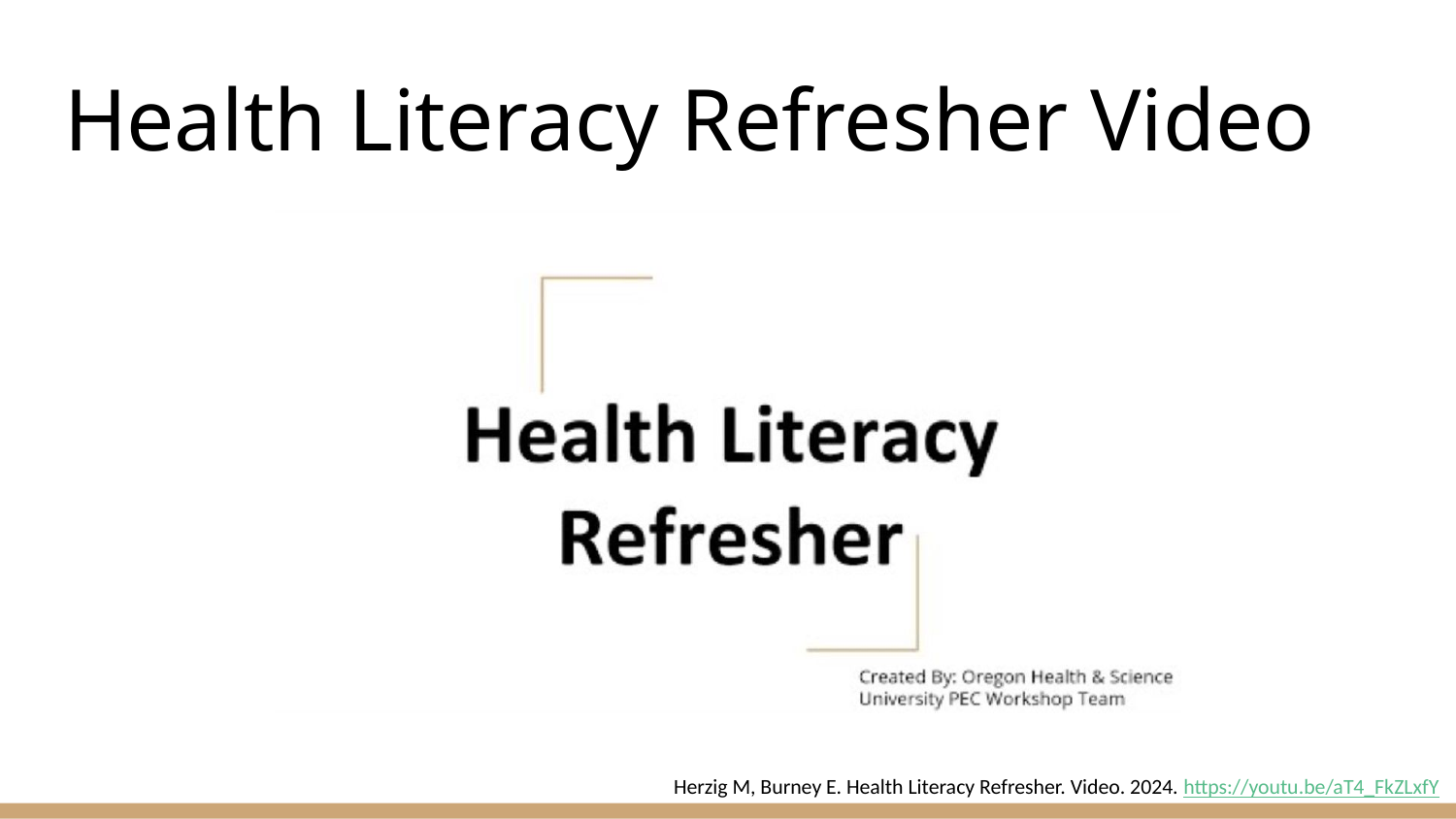

# Health Literacy Refresher Video
Herzig M, Burney E. Health Literacy Refresher. Video. 2024. https://youtu.be/aT4_FkZLxfY

## Slide 5
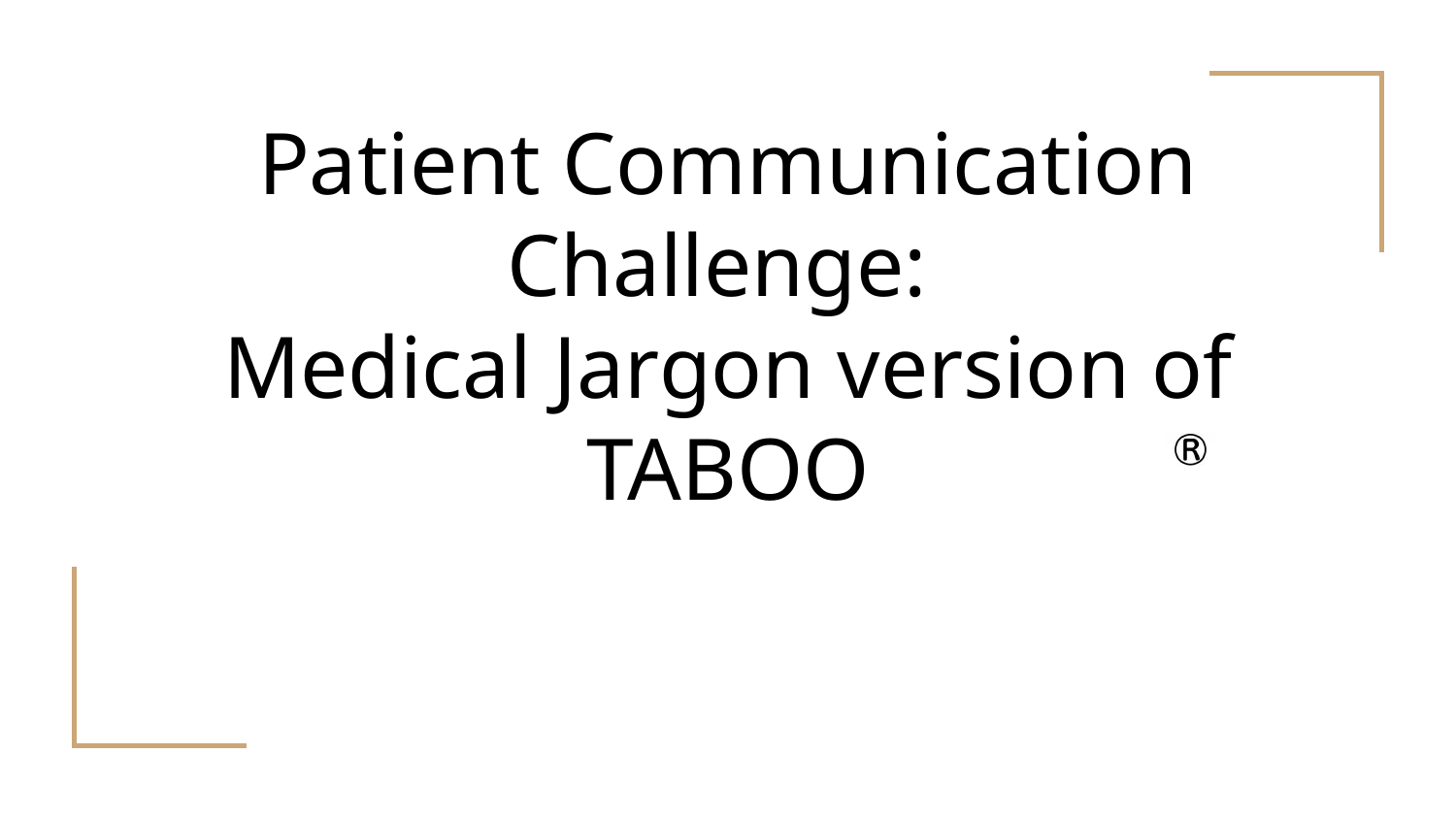

# Patient Communication Challenge: Medical Jargon version of TABOO

## Slide 6
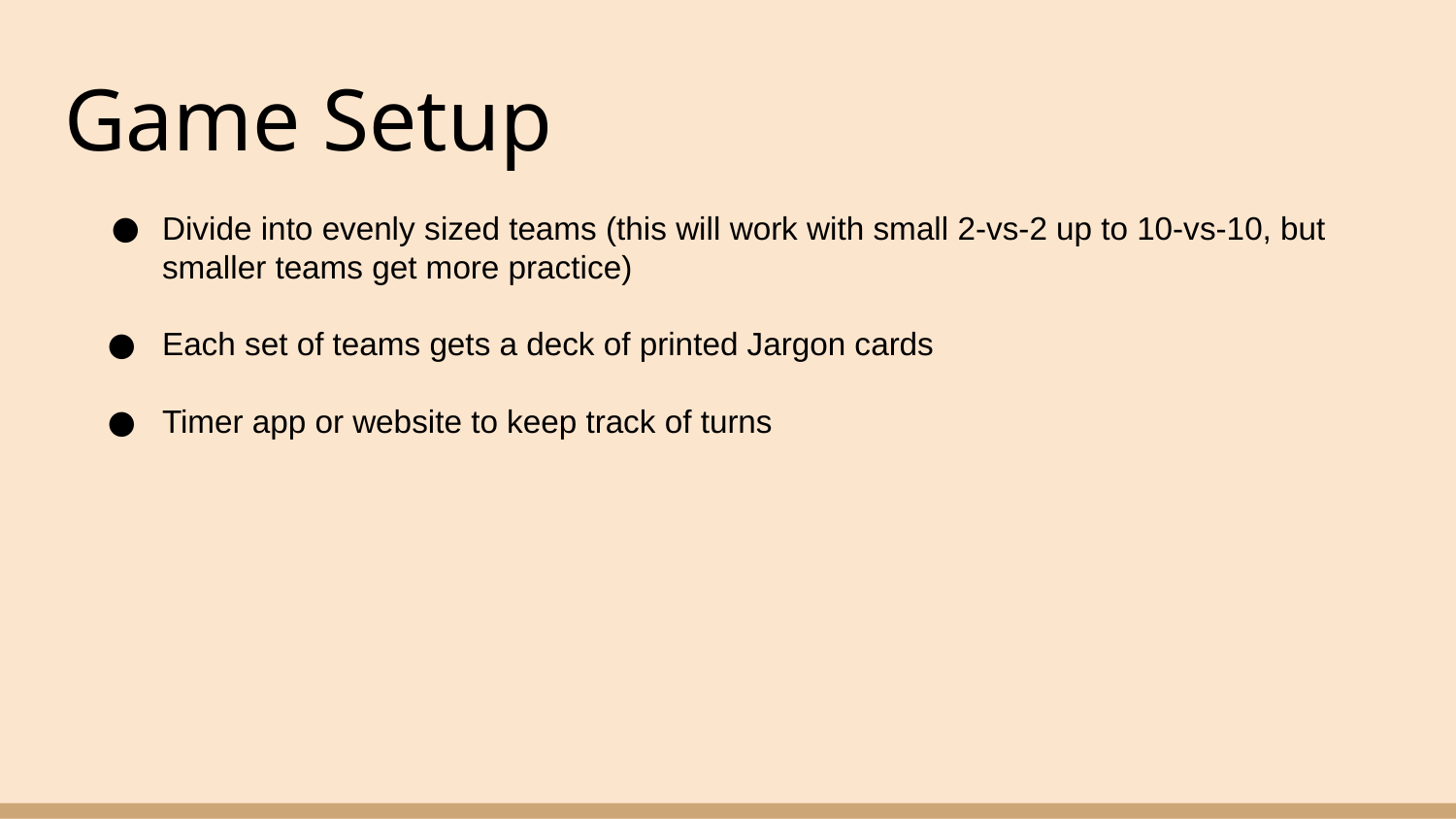

# Game Setup
Divide into evenly sized teams (this will work with small 2-vs-2 up to 10-vs-10, but smaller teams get more practice)
Each set of teams gets a deck of printed Jargon cards
Timer app or website to keep track of turns

## Slide 7
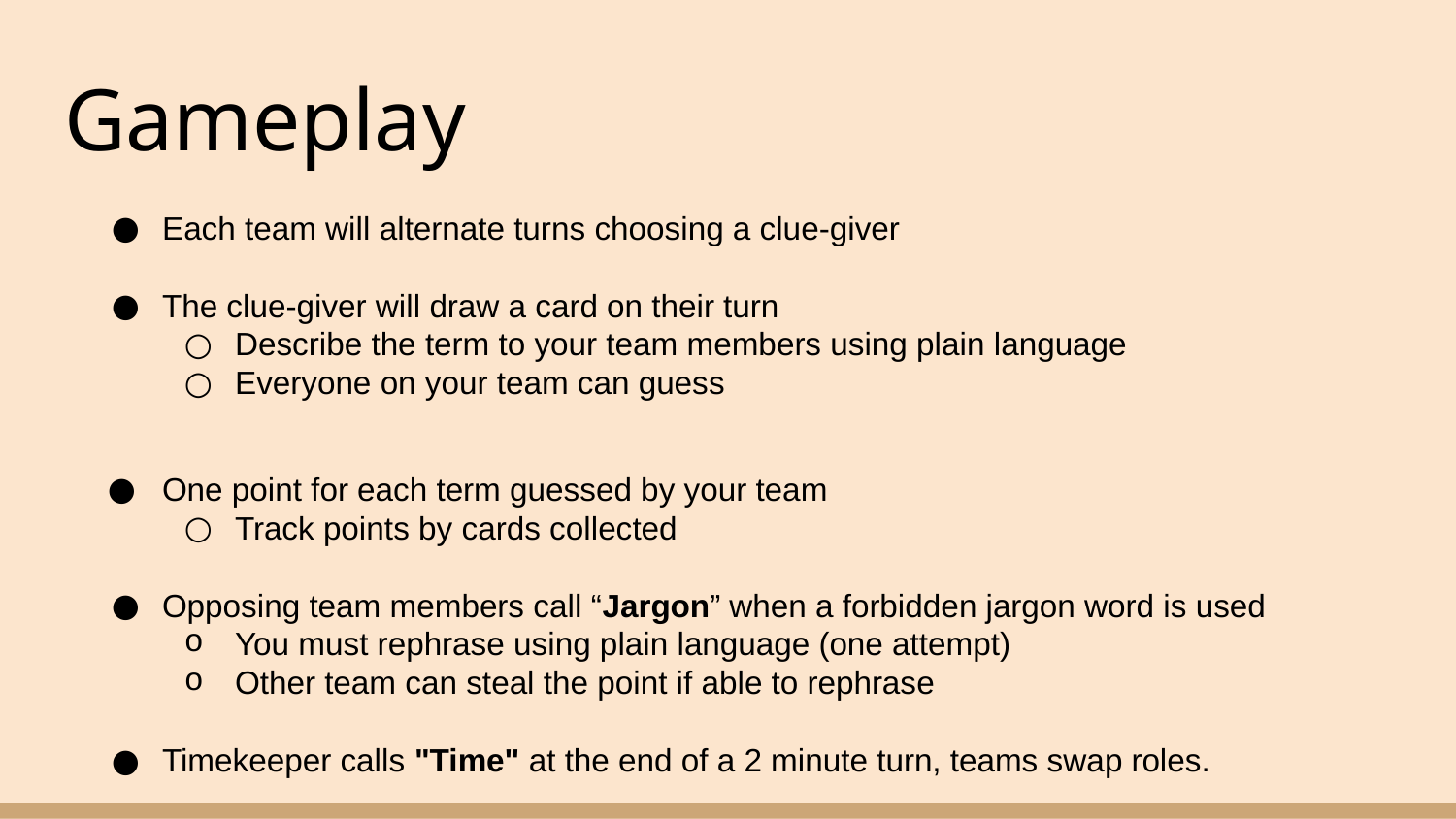

# Gameplay
Each team will alternate turns choosing a clue-giver
The clue-giver will draw a card on their turn
Describe the term to your team members using plain language
Everyone on your team can guess
One point for each term guessed by your team
Track points by cards collected
Opposing team members call “Jargon” when a forbidden jargon word is used
You must rephrase using plain language (one attempt)
Other team can steal the point if able to rephrase
Timekeeper calls "Time" at the end of a 2 minute turn, teams swap roles.

## Slide 8
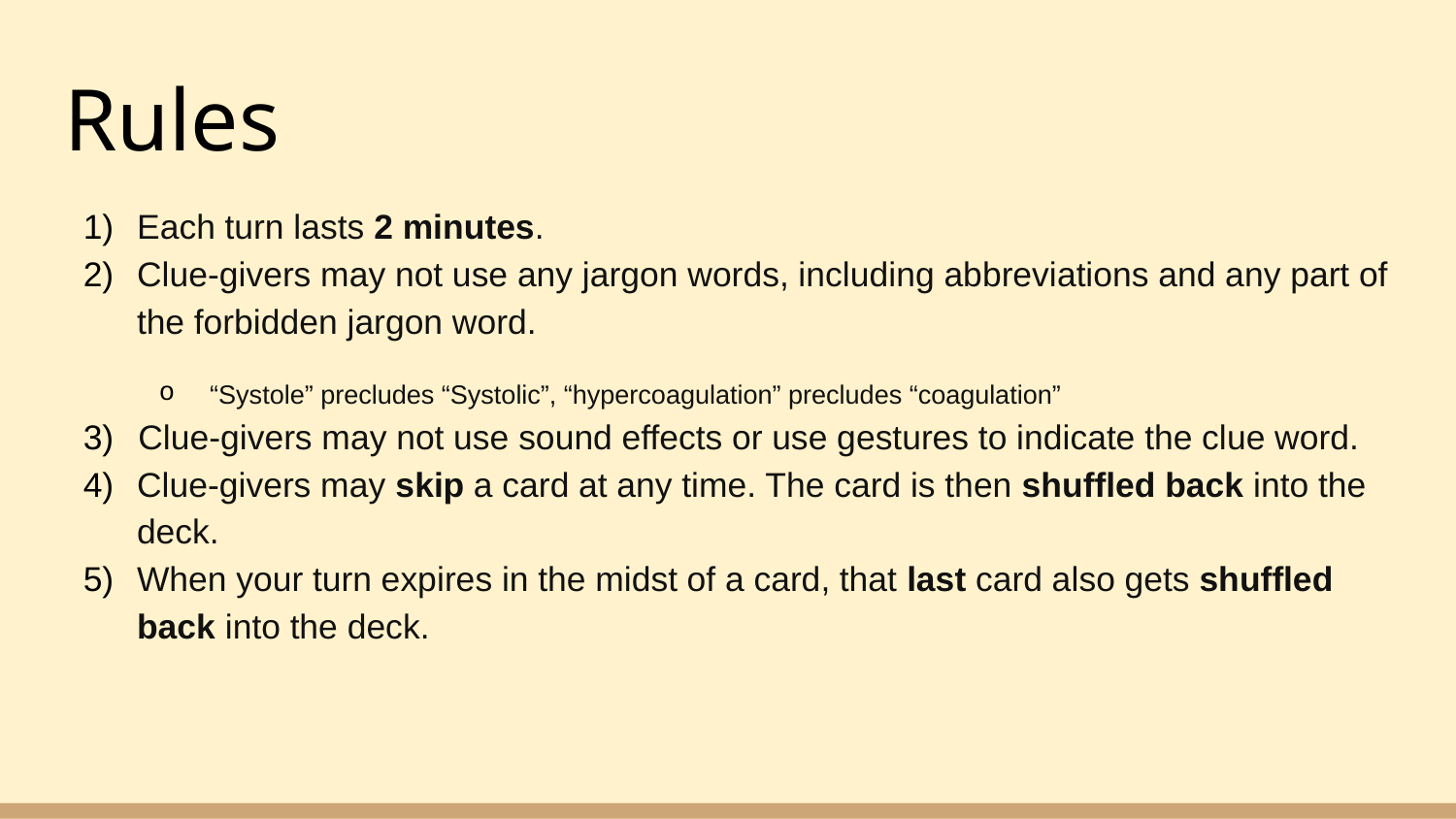

# Rules
Each turn lasts 2 minutes.
Clue-givers may not use any jargon words, including abbreviations and any part of the forbidden jargon word.
“Systole” precludes “Systolic”, “hypercoagulation” precludes “coagulation”
Clue-givers may not use sound effects or use gestures to indicate the clue word.
Clue-givers may skip a card at any time. The card is then shuffled back into the deck.
When your turn expires in the midst of a card, that last card also gets shuffled back into the deck.

## Slide 9
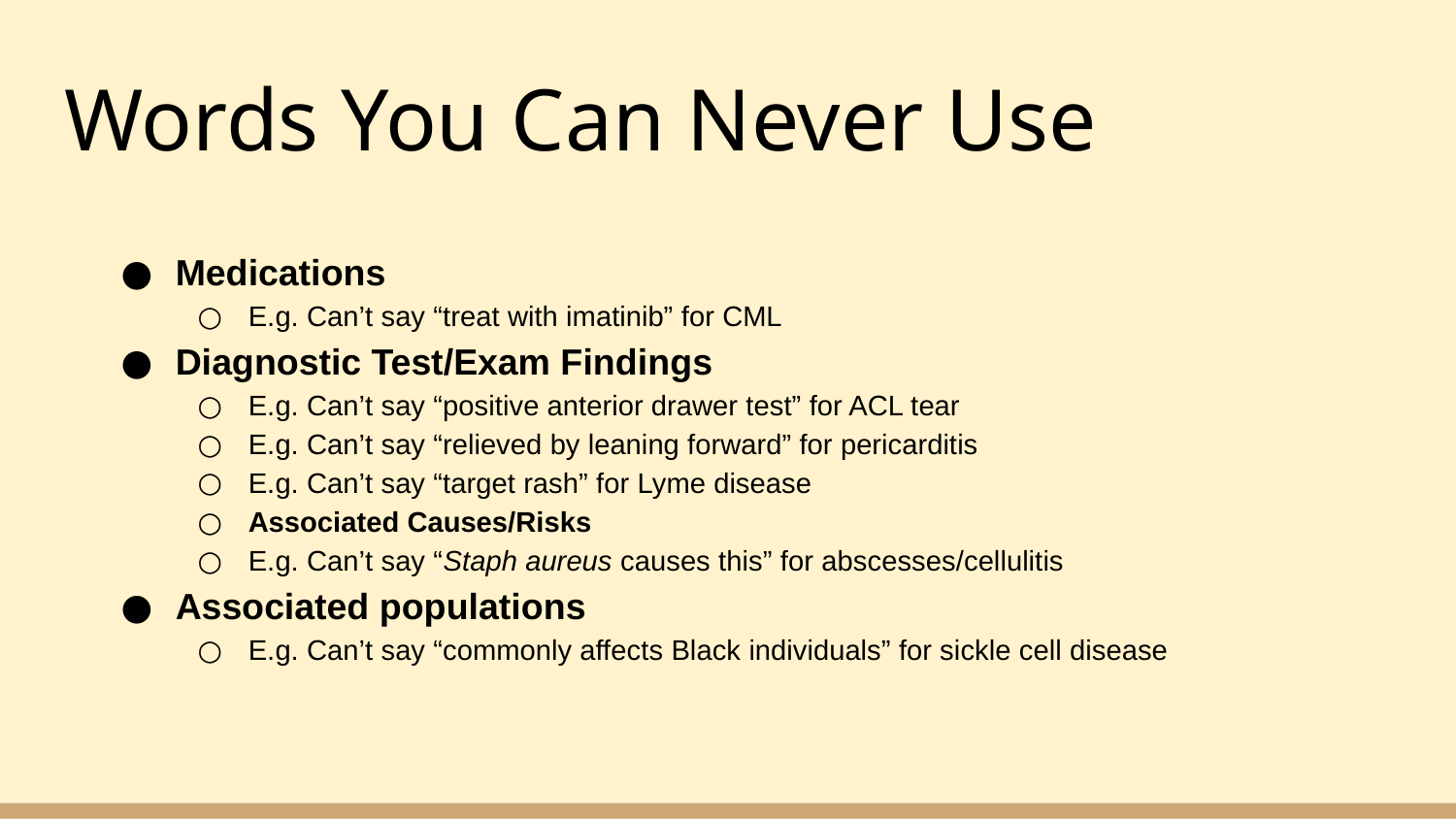

# Words You Can Never Use
Medications
E.g. Can’t say “treat with imatinib” for CML
Diagnostic Test/Exam Findings
E.g. Can’t say “positive anterior drawer test” for ACL tear
E.g. Can’t say “relieved by leaning forward” for pericarditis
E.g. Can’t say “target rash” for Lyme disease
Associated Causes/Risks
E.g. Can’t say “Staph aureus causes this” for abscesses/cellulitis
Associated populations
E.g. Can’t say “commonly affects Black individuals” for sickle cell disease

## Slide 10
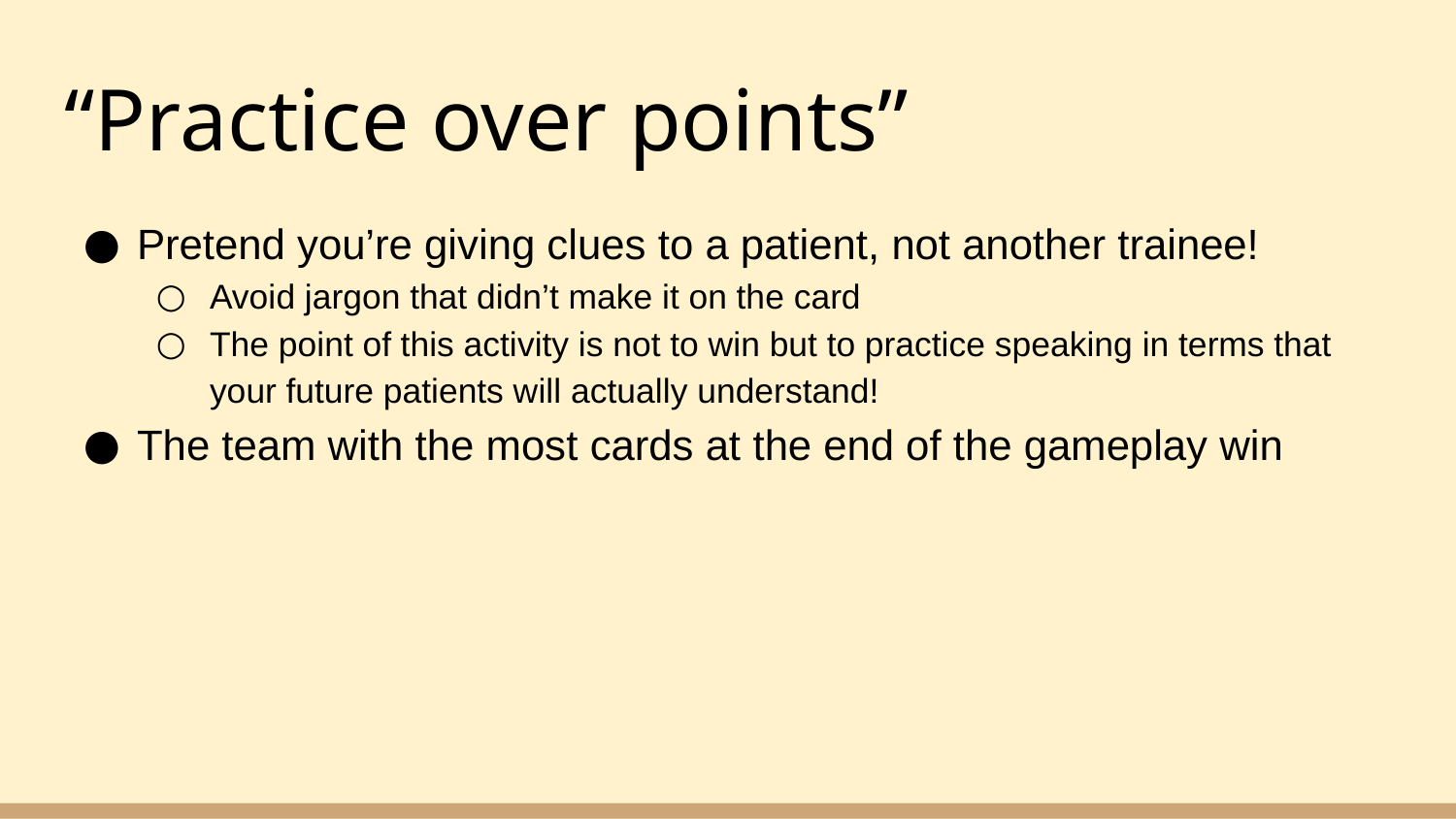

# “Practice over points”
Pretend you’re giving clues to a patient, not another trainee!
Avoid jargon that didn’t make it on the card
The point of this activity is not to win but to practice speaking in terms that your future patients will actually understand!
The team with the most cards at the end of the gameplay win

## Slide 11
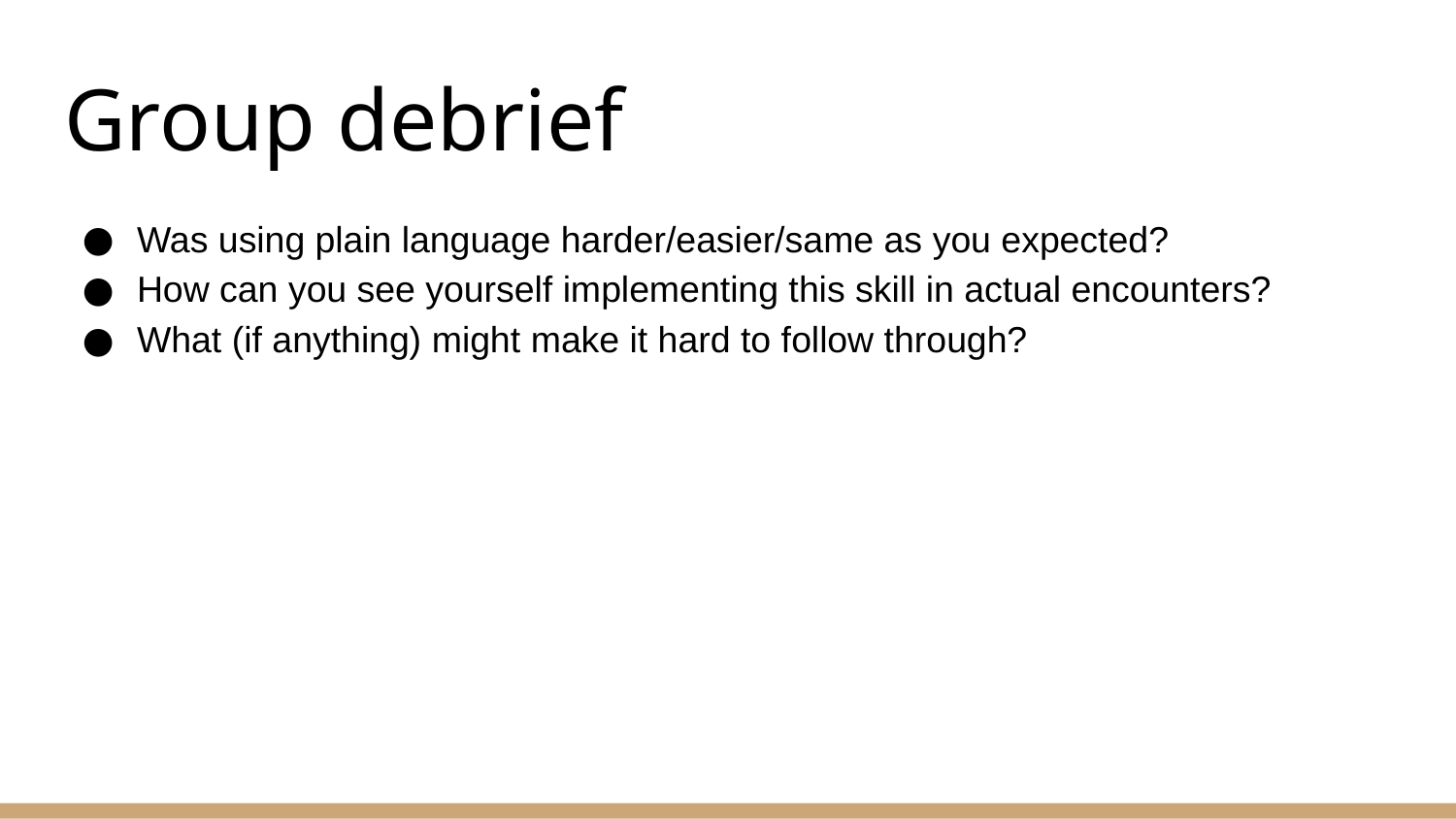

# Group debrief
Was using plain language harder/easier/same as you expected?
How can you see yourself implementing this skill in actual encounters?
What (if anything) might make it hard to follow through?

## Slide 12
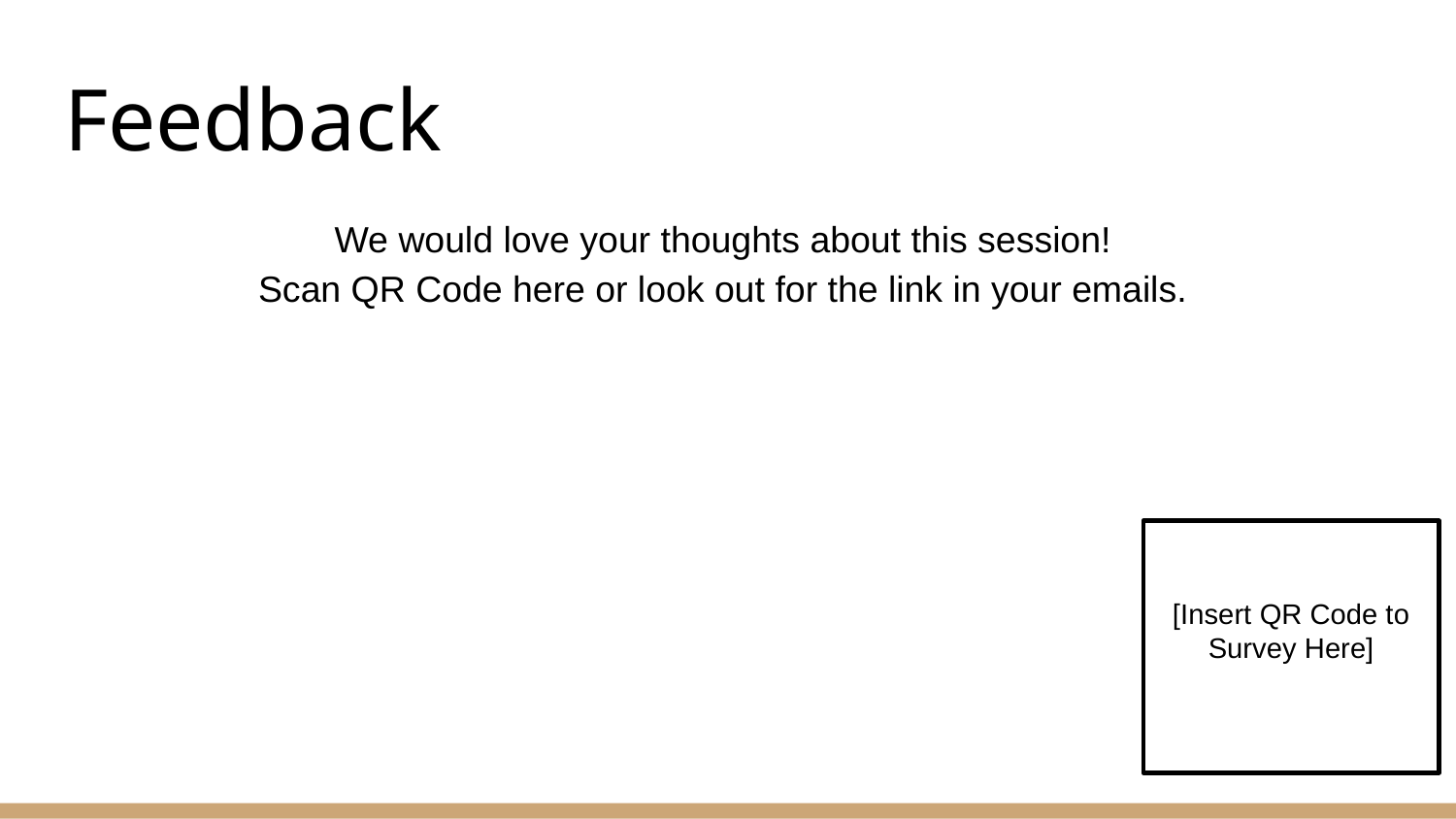

# Feedback
We would love your thoughts about this session!
Scan QR Code here or look out for the link in your emails.
[Insert QR Code to Survey Here]
